# Integrating Murine Gene Expression Studies to Understand Obstructive Lung Disease Due to Chronic Inhaled Endotoxin

**DOI:** 10.1371/journal.pone.0062910

**Published:** 2013-05-13

**Authors:** Peggy S. Lai, Oliver Hofmann, Rebecca M. Baron, Manuela Cernadas, Quanxin Ryan Meng, Herbert S. Bresler, David M. Brass, Ivana V. Yang, David A. Schwartz, David C. Christiani, Winston Hide

**Affiliations:** 1 Massachusetts General Hospital, Boston, Massachusetts, United States of America; 2 Harvard School of Public Health, Boston, Massachusetts, United States of America; 3 Brigham and Women’s Hospital, Division of Pulmonary and Critical Care Medicine, Boston, Massachusetts, United States of America; 4 Battelle, Columbus, Ohio, United States of America; 5 Department of Pediatrics/Neonatology, Duke University Medical Center, Durham, North Carolina, United States of America; 6 Center for Genes, Environment and Health, National Jewish Health, Denver, Colorado, United States of America; 7 Department of Medicine, University of Colorado, Denver, Aurora, Colorado, United States of America; Boston University Medical Center, United States of America

## Abstract

**Rationale:**

Endotoxin is a near ubiquitous environmental exposure that that has been associated with both asthma and chronic obstructive pulmonary disease (COPD). These obstructive lung diseases have a complex pathophysiology, making them difficult to study comprehensively in the context of endotoxin. Genome-wide gene expression studies have been used to identify a molecular snapshot of the response to environmental exposures. Identification of differentially expressed genes shared across all published murine models of chronic inhaled endotoxin will provide insight into the biology underlying endotoxin-associated lung disease.

**Methods:**

We identified three published murine models with gene expression profiling after repeated low-dose inhaled endotoxin. All array data from these experiments were re-analyzed, annotated consistently, and tested for shared genes found to be differentially expressed. Additional functional comparison was conducted by testing for significant enrichment of differentially expressed genes in known pathways. The importance of this gene signature in smoking-related lung disease was assessed using hierarchical clustering in an independent experiment where mice were exposed to endotoxin, smoke, and endotoxin plus smoke.

**Results:**

A 101-gene signature was detected in three murine models, more than expected by chance. The three model systems exhibit additional similarity beyond shared genes when compared at the pathway level, with increasing enrichment of inflammatory pathways associated with longer duration of endotoxin exposure. Genes and pathways important in both asthma and COPD were shared across all endotoxin models. Mice exposed to endotoxin, smoke, and smoke plus endotoxin were accurately classified with the endotoxin gene signature.

**Conclusions:**

Despite the differences in laboratory, duration of exposure, and strain of mouse used in three experimental models of chronic inhaled endotoxin, surprising similarities in gene expression were observed. The endotoxin component of tobacco smoke may play an important role in disease development.

## Introduction

Endotoxin (or lipopolysaccharide, LPS) is a cell-wall component of gram-negative bacteria and is ubiquitous in the environment. Endotoxin has been detected in household dust at low or moderate concentrations [1], and at much higher concentrations in occupational settings such as in swine farms, poultry houses, sewage treatment plants, humidified buildings, and processing of organic materials - in particular cotton [2]. The relationship between endotoxin exposure and the development of asthma is conflicting, with studies suggesting a protective effect of endotoxin in early childhood exposure on the development of asthma [3] while later exposure suggest that endotoxin exposure is associated with both asthma diagnosis and severity [1]. Studies in cotton textile workers have demonstrated the development of an asthma-like syndrome with reversible airflow obstruction termed byssinosis after several years of exposure, while longitudinal studies have demonstrated that with decades of exposure there is an accelerated decline in lung function consistent with chronic obstructive lung disease (COPD), even in the absence of cigarette smoke exposure [4]. More broadly, exposure to biomass fuel has been cited as a major cause of non-tobacco related obstructive lung disease, with roughly 3 billion people exposed worldwide [5], [6]. While prior studies on biomass fuel and COPD have focused on the role of particulate matter a recent study noted high levels of airborne endotoxin (up to 365 EU/m^3^) in homes burning biomass fuel, with higher endotoxin levels noted in less processed solid fuels such as dried animal dung [7]. Intriguingly, bioactive LPS has also been detected in cigarette smoke, and it has been estimated that the amount of endotoxin delivered from smoking one pack of cigarettes a day is equivalent to that experienced daily by cotton textile workers at risk for byssinosis [8].

The mechanisms whereby endotoxin might protect against, or lead to obstructive lung disease remain unclear, and the phenotype of obstructive lung disease (reversible airflow obstruction in asthma vs. irreversible airflow obstruction in COPD) related to inhaled endotoxin has not been well characterized. A poorly understood and understudied overlap syndrome between asthma and COPD is seen in clinical practice, where patients at times may exhibit reversible airflow obstruction but at other times might present with irreversible airflow obstruction [9].

While most animal models of COPD utilize inhaled endotoxin as a model for acute COPD exacerbations rather than for the development of chronic COPD over longitudinal exposure [10], several experimental animal models have demonstrated that long-term exposure to inhaled endotoxin leads to increased airways resistance and hyper-reactivity to methacholine challenge [11]–[14] as well as histologic evidence of emphysema [15], [16] and airway narrowing associated with fibroproliferation [17]. Several groups have used microarray technology to characterize the pulmonary gene expression profile associated with chronic inhaled endotoxin. Airflow obstruction has been noted in these models [15], [17]–[19], and pulmonary gene expression profiling has demonstrated over-expression of genes such as serum amyloid A 3(Saa3), matrix metalloproteinase 12 (MMP-12), and lymphocyte antigen 6 complex locus I (Ly6i), a cell surface marker with unknown function found on the surface of T and immature B cells.

However, to date no studies have examined the agreement between model systems at the gene or pathway level. Microarray data generated in different laboratories may vary greatly based on strain of mouse and endotoxin used, different exposure protocols, and different array platforms [20], [21]. In these situations, confirmation of findings based on agreement between results from other groups represents an important method of validation [22].

In this study we hypothesized that a combined analysis of gene expression microarray data sets from all available experimental murine models of chronic inhaled endotoxin would identify a shared, robust signature at both the gene and pathway level. We further hypothesized that this signature would yield biologic insight into the phenotype of endotoxin-related obstructive lung disease as well as potential dysregulated pathways. Finally, to assess the biological significance of this endotoxin signature in complex endotoxin containing exposures such as cigarette smoke, we used the endotoxin signature that we identified to accurately classify mice exposed to either smoke alone, endotoxin alone, or smoke and endotoxin.

## Materials and Methods

Please refer to **Figure S1** for an overview of the methods.

### Identification of Studies for Inclusion

A murine model of chronic exposure to inhaled endotoxin was developed in our laboratory [23]. A thorough literature search was conducted in order to identify all additional published studies where the study design included murine models of repeated inhaled endotoxin exposure with extraction of RNA from lung homogenate for microarray analysis. A computer search of PubMed with the following search terms “Gene Expression Profiling”[Mesh]) AND “Lipopolysaccharides”[Mesh] AND :Lung:[Mesh] to identify candidate studies and also hand-searched references in the articles. Four experimental models of endotoxin exposure from three distinct laboratories were identified, two from the same laboratory [18], [19], [23]. The raw gene expression data was obtained from authors through written correspondence.

### Normalization and Data Analysis

To ensure consistent processing and annotation of results, re-analysis of all samples was performed using BioConductor/R version 2.13 (www.bioconductor.org) for Affymetrix arrays and the TM4 Microarray Software Suite (http://www.tm4.org/) for Agilent arrays.

For arrays performed on the Affymetrix platform, the quality of the microarray analysis was confirmed using the arrayQualityMetrics package [24]. Background adjustment, quantile normalization, and summarization was performed with RMA using the simpleaffy package [25]. Using the siggenes package, pairwise analysis was performed with significance analysis of microarrays (SAM) [26] to identify statistically significant changes in gene expression. The delta was chosen to limit the output gene list to a false discovery rate (FDR) of less than 5%.

### Identification and Validation of Gene Signature

The gene signature for chronic inhaled endotoxin was defined by a common intersect of differentially regulated genes across 3 experiments. As the genes interrogated by each platform differed, each probeset from each platform was mapped to both common MGI gene symbols and common Entrez gene identifiers in order to identify a common intersect of both differentially expressed genes as well as genes interrogated by each microarray platform to identify a common background distribution of genes. As there is no readily available implementation for 3-way hypergeometric tests, 2-way hypergeometric tests were performed to test for the statistical significance of the overlap between any two studies.

### Comparison of Studies at the Gene and Pathway Level

Annotation of each gene in the gene signature was performed. For each study, genes with significant differential expression were tested for enrichment in pathways from KEGG [27], WikiPathways [28], Reactome [29], and Netpath [30] using a hypergeometric test (p≤0.05). The twenty most significant GO terms from each study were merged into a single representation as long as they reached significance in at least one study and visualized as a functional network using Cytoscape [31]. To identify other experimental studies that have differentially expressed genes highly correlated with the identified endotoxin gene signature, we used Nextbio. Nextbio is a proprietary software program that aggregates all publicly available high-throughput microarray data from repositories such as Gene Expression Omnibus (GEO), and performs quality control and significance testing to identify differentially expressed genes.

The system supports the calculation of pairwise gene signature correlation scores using rank-based enrichment statistics between a user-provided input gene signature and ranked gene lists generated from the public data sets within the NextBio corpus [32]. We identified the top 20 experimental conditions with the highest enrichment scores based on the endotoxin gene signature.

### Interrogating Biological Significance of Endotoxin Gene Signature in Tobacco Exposure

Consensus clustering [33] using expression probe intensity values was used to assess the role of the endotoxin-associated gene signature in smoking related lung disease. In a study in which mice were exposed to air (as a control), LPS alone, smoke alone, and smoke as well as LPS, consensus clustering was performed by subsampling the gene signature (80% of the gene signature, repeated 1000 times) and assessing pairwise consensus values, the proportion that two items occupied the same cluster out of the number of times they occurred in the same subsample. The consensus values were compared to the mean consensus clustering value of 1000 random gene signatures of the same size.

## Results

Four studies from three different investigators were identified from our literature search [18], [19], [23]. Characteristics of each study are as detailed in **Table 1**. There were differences between these studies in the strain of LPS and mouse used as well as the exposure protocol, microarray platform, and lab where the study was conducted. Using a FDR cutoff of 5%, 578, 3083, and 2256 genes were found to be differentially expressed for the Lai (5 day), Meng, and Brass datasets, respectively.

**Table 1:** Description of all included studies including differences by endotoxin strain, mouse strain, exposure protocol, and microarray platform used.

Investigator	Endotoxin strain	Mouse strain	Exposure protocol	Microarray platform
Brass et al [18]	*Escherichia coli*	C57BL/6	5 µg/m^3^ atomized LPS with air as control	Agilent 20K customized
	serotype		4 hrs/day, 5 days/week	
	0111:B4		1 week duration	
			N = 8 per exposure group^a^	
Meng et al [19]	*Escherichia coli*	AKR/J	0.5 µg/L nebulized LPS with air as control	Affymetrix mouse genome 430 2.0
	serotype		1 h/day, 2×/week	
	O55:B5		3 week duration	
			N = 6 for air exposure, N = 5 for LPS exposure	
Lai et al [23]	*Pseudomonas aeruginosa*	C57BL/6	2mg/day nebulized LPS with nebulizedphosphate buffered saline (PBS) as control	Affymetrix mouse genome 430a 2.0
	serotype 10		15 min/day, 5 days/week	
			5 day duration^b^	
			N = 4 per exposure group	
			2 mg/day nebulized LPS with nebulizedphosphate buffered saline (PBS) as control	
			15 min/day, 5 days/week	
			8 week duration^b^	
			N = 4 per exposure group	

a In this study, 24 mice were exposed to each condition, with RNA was pooled 3 mice per array, for a total of 8 arrays per experimental condition,

b 5 day duration used as part of training set to identify common gene signature, 8 week duration used as test set to determine if gene signature can accurately classify between endotoxin and control phosphate buffered saline (PBS) exposed mice.

**Figure 1:**
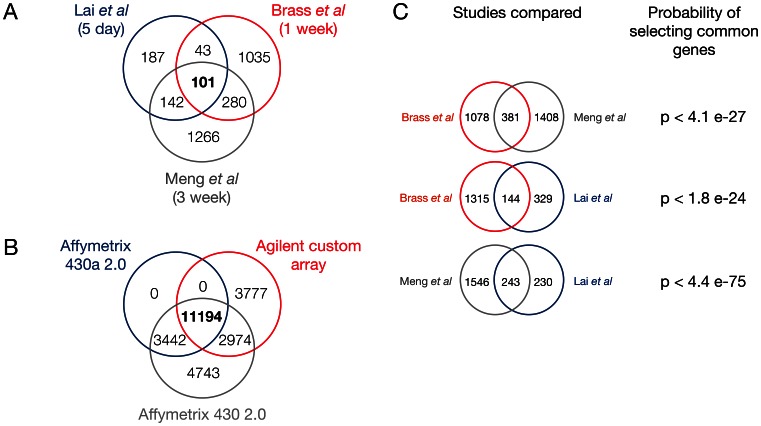
Comparison of 3 identified studies of murine inhaled endotoxin exposure used to generate gene signature. **1a.** Common intersect of differentially regulated genes (endotoxin vs. control exposure) identifies a gene signature for endotoxin exposure. **1b**. Common intersect of arrays used in each studies represents a background distribution to identify the statistical significance of the gene signature. **1c**. 2-way hypergeometric tests to identify statistical significance of gene signature.

101 genes were found to be differentially expressed in common across all three studies (**Figure 1a** **, Table S1**). Comparison of the genes mapped by each array revealed that 11,194 genes were present in the array platform across all three studies (**Figure 1b**). As there is no readily available method to implement 3-way hypergeometric tests, 2-way hypergeometric tests were performed to detect the statistical significance of the overlap between differentially expressed genes in each study. P-values were less than 4.1×10^−27^, 1.8×10^−24^, and 4.4×10^−75^ when comparing Brass vs Meng, Brass vs Lai, and Meng vs Lai (**Figure 1c**).

The genes present in the 101-gene signature are as listed in **Table 2**. As internal validation, we looked for the presence of proteins encoded by genes present in the well described LPS signaling pathway [34]. Both *lbp* (LPS binding protein) and *cd14* (cluster of differentiation 14) are present, although *tlr4* (Toll-like receptor 4) and *ly96* (lymphocyte antigen 96, which codes for the protein MD-2) were not. Further validation of the gene signature was performed by confirming that these 101 genes could accurately classify a separate 4^th^ endotoxin and control PBS exposed murine experiment (Lai 8 week model, **Figure S2**).

**Table 2:** Mouse genome identifier (MGI) gene symbols for all genes identified in the 101 gene signature.

1100001G20Rik	Ch25h	Fn1	Ly6i	Rab32
Acp2	Chi3l1	Fpr2	Matn4	Reg3g
Atp6ap2	Chi3l3	Gatm	Mmp12	Rmcs2
B4galnt1	Clec4a2	Grn	Ms4a6d	Saa3
Bcl2a1a	Clec4n	H2-Ab1	Ms4a7	Sirpa
Bcl2a1b	Clec7a	Havcr2	Mtm1	Slc26a4
Bcl2a1d	Clu	Hvcn1	Muc1	Slc3a2
Bst1	Cp	Id2	Naip1-rs1	Slc6a20a
C1qb	Csf2rb2	Ifi30	Naip2	Smpdl3b
C1ra	Ctsb	Ifit3	Olfm1	Snx10
C1rb	Ctsk	Igf1	Olr1	Tgfbi
C2	Ctss	Il1rn	Orm1	Tgfbr1
C3	Ctsz	Il33	Orm2	Tifa
Capg	Cxcl17	Itgax	Per3	Tlr7
Ccl6	Cxcl2	Itgb2	Pigr	Tmem106a
Ccl9	Cyba	Itih4	Pon1	U46068
Cd14	Cybb	Lair1	Prkcd	Vnn1
Cd1d1	Dab2	Laptm5	Procr	
Cd200r1	Dbp	Lbp	Psap	
Cd68	Emr1	Lgals3bp	Ptgs1	
Cfb	F10	Lrg1	Rab20	

Functional evaluation of the 101-gene signature was performed. First, annotation of the 101 genes by querying PubMed to determine an association between these genes and published manuscripts on asthma and COPD revealed a significant amount of overlap, with 1024 asthma related publications and 437 COPD related publications (**Tables S2 and S3**). Gene enrichment analysis was performed using DAVID. The Gene Ontology Biological Processes most enriched were response to wounding, inflammatory response, and acute inflammatory response. Using a repository of published experimental results (NextBio [35]), the 101 gene signature was found in a high frequency of murine asthma experiments (**Table 3**).

**Table 3:** Identifying other studies with similar expression of 101 gene signature in the context of the public corpus of gene expression studies using NextBio.

Study Name	GEO ID
Lung gene expression profiles in a mouse model of IL-13-induced allergic airway inflammation	GSE35979
Lungs of BALB/c wildtype or Rag deficient mice exposed to ovalbumin as an experimental asthma model	GSE6858
Murine pulmonary responses to ambient Baltimore particulate matter	GSE9465
Lungs from IFNg−/−, IRF1−/−, or WT mice infected with M. avium	GSE11809
Effect of a disease-associated human IL-4 receptor allele in experimental asthma	GSE18010
Hookworm-Induced Persistent Changes to the Immunological Environment of the Lung	GSE5555
Virus-Induced Airway Disease in Mice	GSE10964
The effect of IL-13 and dust mites on gene expression in murine model of asthma	GSE1301
Lungs of BAL/C mice sensitized with ovalbumin (OVA) and exposed to diesel exhaust particles (DEP)	GSE22357
Lungs of C57BL6 mice infected with a low dose of M. tuberculosis for 30 and 70 days	E-MEXP-1899
Lungs from mice in time course infection study of pandemic H1N1 influenza A isolate A/CA/4/2009	GSE37569
Murine Airway Hyperresponsiveness	GSE3184
Hyperlipidemic aorta atherosclerosis in ApoE null mice	GSE21419
Immune response to Pneumocystis Infection in WT and CD40 Ligand Deficient Mice	GSE11005
Lung expression in Foxa3 knock-out and wildtype mice challenged with allergen	GSE13382
Plasma cell tumor progression	GSE34078
Lungs from mice exposed to bleomycin	GSE16846
Ovalbumin sensitized and challenged A/J mice	GSE450
Lung gene expression in ovalbumin (OVA)-induced experimental asthma	GSE11911
Mndal suppresses cell growth and may modify plasmacytoma susceptibility	GSE17297

The top 20 experiments with gene signatures present in the NextBio database that have the highest enrichment scores for the 101 gene signature are listed below. Multiple murine asthma studies demonstrate similar gene expression patterns to the 101 gene signature.

**Figure 2:**
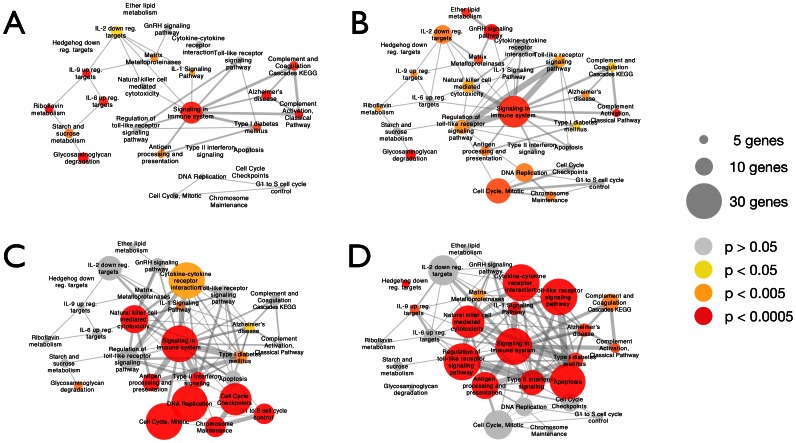
Comparison of all 4 identified studies at the pathway level. Pathway enrichment calculated using hypergeometric tests with all known pathways present in Netpath, Wikipathways, Kegg, and Reactome, with Cytoscape for visualization. **2a.** Lai et al, 5 day exposure. **2b**. Lai et al, 8 week exposure. **2c**. Meng et al, 3 week exposure. **2d**. Brass et al, 1 week exposure.

A pathway based comparison of the 3 experiments was performed using all known pathways present in Netpath, Wikipathways, Kegg, and Reactome. We used hypergeometric tests to determine pathway enrichment from each experiment and visualized the results with Cytoscape (**Fig. 2**). Multiple similarities at the pathway level were noted, including in the complement, coagulation, and cell adhesion pathways. Within the same experimental model [23], longer duration of LPS exposure (Lai et al 5 day vs. 8 week) was associated with an increased enrichment of inflammatory pathways (**Fig. 2a** vs. **Fig. 2b**).

**Figure 3:**
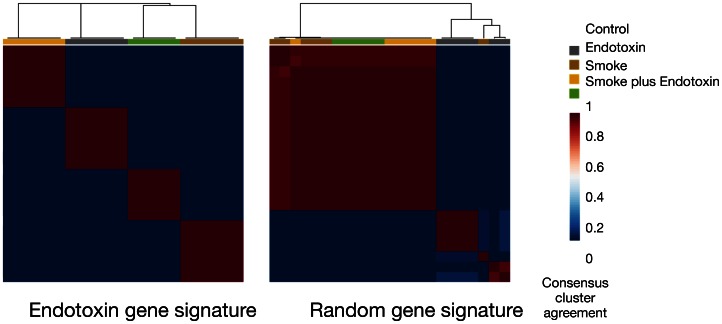
Consensus clustering of air, endotoxin, smoke, and endotoxin plus smoke exposed mice using endotoxin gene signature and random gene signature. The endotoxin gene signature accurately clusters the different exposure groups 99.97% of the time as compared to a randomly chosen gene signature which accurately clusters the different exposure groups 78.15% of the time.

Finally, endotoxin is rarely if ever present as an environmental exposure alone but rather is typically present in conjunction with other exposures. Endotoxin is a known component of tobacco smoke [8], and so we sought to determine whether the key genes involved in the response to endotoxin are also important in the response to tobacco smoke. In experiments performed at a single laboratory where mice were exposed to air (as control), endotoxin, tobacco, and endotoxin with tobacco [19] (**Table 4**), and gene expression profiling was performed on lung homogenate, the 101 gene signature accurately classified endotoxin vs. smoke vs. endotoxin plus smoke exposed mice (**Figure S3**). To assess the stability of the classification as well as to determine whether accurate classification was likely due to chance, we used consensus clustering where the ability of the endotoxin gene signature to accurately classify 1000 bootstrapped samples from the data was compared to a randomly chosen gene signature of equal size. Consensus clustering by the gene signature accurately classified air vs. endotoxin vs smoke vs smoke plus endotoxin exposed groups 99.97% of the time based on 1000 randomly chosen subsamples of probes from all arrays, vs. 78.15% of the time using an equal number of randomly chosen genes (**Figure 3**). This suggests that the ability of the endotoxin gene signature to classify between these subgroups is better than a random selection of genes.

**Table 4:** Description of exposure protocol used in comparing endotoxin, cigarette smoke, and endotoxin plus cigarette smoke exposed mice.

Investigator	Endotoxin strain	Mouse strain	Exposure protocol	Microarray platform
Meng et al [19]	*Escherichia coli*	AKR/J	**Air only (Control)**	Affymetrix mouse
			HEPA-filtered air	genome 430 2.0
	serotype		3 weeks	
	O55:B5		N = 6	
			**LPS only**	
			0.5 µg LPS/L nebulized LPS1 h/day, 2x/week	
			3 weeks	
			N = 5	
			**Smoke only**	
			2R4F cigarettes (250 µg WTPM) 5 h/day, 5x/week	
			3 weeks	
			N = 6	
			**LPS+smoke**	
			Smoke protocol (5x/week) with concurrent LPS protocol (2x/week)	
			3 weeks	
			N = 5	

## Discussion

In this study, we made a number of novel observations. First, we identified a common set of 101 genes that was differentially regulated in endotoxin exposed vs. control (PBS or air exposed) mice across all published models of recurrent endotoxin exposure. A number of genes previously identified as being important in either the pathogenesis or severity of COPD and asthma were present in the signature. In addition, there are a number of other genes identified not previously associated with obstructive lung disease, and may represent candidate genes for further investigation. Of particular interest is the ability of this 101 gene signature to accurately classify endotoxin, smoke, and endotoxin plus smoke exposed mice. While there appear to be some similarities in differential gene expression in response to endotoxin, to cigarette smoke, or to cigarette smoke in conjunction with endotoxin, whether this translates into a similar phenotype of obstructive lung disease cannot be concluded from this observation.

Comparison of these studies at the pathway level revealed additional similarities across these experiments. Notably, when looking within the same experimental model, longer duration of endotoxin exposure led to increased enrichment of inflammatory pathways, which is in contrast to prior studies suggesting that endotoxin tolerance develops in response to repeated endotoxin challenge [36]. While development of tolerance may be related to a number of factors such as the method or route of exposure, it deserves further study as chronic inflammation is thought to play an important role in both asthma and COPD.

While the 101 genes identified represents a small number of genes that are differentially expressed across experimental models as compared to the number of genes differentially expressed within any of the identified experimental models, factors that have previously been identified as being important in the biological response to endotoxin and likely contributed to the heterogeneity in response include significant differences in strain of endotoxin, strain of mouse, exposure protocol, and local practices of each lab, and likely contributed to differentially expressed genes that were not conserved across experimental models [37], [38]. We evaluated the significance of these 101 genes on a number of levels. First, using hypergeometric tests, we demonstrated that this common intersect is highly unlikely due to chance alone. Second, examination of the 101 genes revealed the presence of *lbp and cd14,* which are well described components of the endotoxin signaling pathway, thus affirming the biologic relevance of this gene signature. Third, this gene signature was able to accurately classify between endotoxin and phosphate buffered saline exposed mice in a distinct experiment not used to generate the gene signature. Finally, at the pathway level, we observed that increasing duration of endotoxin exposure led to increasing enrichment of inflammatory pathways; this was confirmed in a recent publication from our group [23], where increasing duration of inhaled endotoxin exposure was associated with increased IL-6 and decreased IL-10 concentrations in lung homogenate, consistent with a persistent pro-inflammatory profile. The increase in inflammation with prolonged endotoxin exposure was associated with and potentially mediated by an expansion of lung dendritic cells and a reduction in macrophages; thus it was not surprising to see that within the 101 gene signature, there were a number of genes important in antigen presentation that were differentially expressed (such as *h2ab-1* which encodes for MHC class II, myeloid chemokines such as *ccl6* and *ccl9,* cathepsins such as *ctsz, ctss, ctsb,* and *psa,* and C-type leptin-like receptors such as *clec4a2, clec4n,* and *clec7a*). Recent human studies have demonstrated the accumulation of dendritic cells in COPD, with an association between disease severity and level of dendritic cell accumulation. [39], [40]On examination of the genes present in the 101 gene signature (**Tables S1 and S2**), a number of asthma associated genes were present, including *chi3l1*, which was identified in one of the first genome-wide association (GWAS) studies of asthma [41], and *il33*, which has been shown in a number of experiments to be important in asthma development [42], [43] and asthma severity [43]. Interestingly, interleukin-33 has also been found to enhance the endotoxin response of macrophages [44]. Several COPD associated genes were present, including *mmp-12,* which has been identified in both murine studies as being associated with the development of emphysema [45] as well as in human studies as being associated with the risk of COPD development in smokers [46]. Of further interest was the identification of *fpr2 and saa3*. Serum amyloid protein (SAA) has previously been considered solely an acute phase reactant, and while *saa1* and *saa2* are expressed primarily in liver and kidney [47], *saa3* is expressed in the lung and has only recently been identified as important in the pathogenesis of glucocorticoid refractory COPD by opposing organ protective signaling by lipoxins at the ALX/FPR2 receptors [48].

While inhaled endotoxin exposure as a model for de novo COPD development rather than COPD exacerbations has received little attention, from epidemiologic studies it is clear that between a quarter to a half of patients with COPD have never smoked [5]. The phenotype of non-tobacco induced COPD as compared to tobacco-related COPD remains poorly studied. The third National Health and Nutrition Examination Survey (NHANES III) has suggested that non-smokers account for 24.9% of COPD cases in the United States [49]; in this study many subjects with non-tobacco COPD previously had a physician diagnosis of asthma. The multi-center, international BOLD study [50] confirms these findings, estimating that between a quarter to a fifth of all patients with COPD are nonsmokers. Indoor biomass fuel exposure and occupational exposure to biologic or organic dusts in the workplace, both of which has been associated with high levels of endotoxin exposure, were associated with non-tobacco COPD. As in the NHANES study, self-reported physician diagnosis of asthma was a strong predictor of non-tobacco COPD. While this may represent disease misclassification by physicians, as the existence of non-tobacco COPD is not widely appreciated, it is also possible that this may relate to the underlying phenotype of non-tobacco COPD that is different from tobacco-related COPD.

The ability of the 101 gene signature to accurately classify between endotoxin, smoke, and endotoxin plus smoke exposed mice, and not just mice exposed to air vs. mice exposed to any endotoxin (whether as endotoxin alone, tobacco smoke [which contains endotoxin], or endotoxin in addition to tobacco smoke) is intriguing. It suggests that genes selected for differential expression between endotoxin and control also play an important role in differential expression between various endotoxin containing exposures. Further examination of expression patterns of the 101 gene signature in this comparison indicate that (**Figure S3**) *chi3l1*, which is associated with asthma, was upregulated only in endotoxin exposed mice. Conversely, *mmp12* was significantly upregulated in all exposure groups although average log_2_ fold change for smoke vs. control was 4.76, for LPS vs. control was 2.05, and for smoke+LPS vs. control was 2.33. MMP-12 does not appear to have an important role in endotoxin induced inflammation [51], but the interaction between smoke and endotoxin has not been well studied. Of note *mmp12* has been associated with the emphysema subtype of COPD [45]. If these gene expression changes are reflected in human exposures and affect downstream clinical phenotypes, it is possible that endotoxin-related COPD has a phenotype more consistent with small airways disease rather than parenchymal disease as seen in tobacco-related emphysema. A further potential implication of the observed differences in *mmp12* expression is that smokers who have recurrent bacterial infections (and thus are exposed recurrently to endotoxin) may be more likely to develop a predominantly airways disease subphenotype of COPD rather than a predominantly emphysema subphenotype of COPD [52].

Strengths of this work include the approach to identify the consistency of gene expression across experimental models, the use of consensus clustering to validate the importance of the identified gene signature against a randomly picked set of genes, and the potential biological applications of the gene signature. This is the first paper to assess the importance of the endotoxin component of cigarette smoke as an exposure using genomic techniques.

We acknowledge that there are several limitations to this work. These studies were performed in murine models and may not be translatable to human disease. Additionally, all of these studies were performed on lung homogenates, and it is difficult to distinguish which cell population contributed to the gene expression signature. Differences between duration of endotoxin exposure were not explicitly addressed as we were looking for agreement across studies; prior work has demonstrated that there are changes in short term vs. long term exposure. [23] Finally, to verify the biologic importance of any one of the identified genes, additional functional work is needed.

To date all treatment trials of COPD have required prior significant tobacco use as an inclusion criteria, and thus we know little about the efficacy of COPD therapies in non-tobacco related COPD. While our work suggests that the endotoxin component of cigarette smoke may be important in disease development, the effect of additional endotoxin in conjunction with tobacco exposure leads to different gene expression changes compared to endotoxin alone. The differentially expressed genes in response to repeated endotoxin exposure that we identified have been implicated in both asthma and COPD, and based on our pathway analysis, chronic inflammation plays a significant role. There may be other biologically-targeted therapies that may have additional benefit in endotoxin-related obstructive lung disease. Prognosis and treatment implications of this disease may or may not differ from tobacco-related COPD, and deserves further study.

## Supporting Information

Figure S1 **Overview of methods.**
(TIF)

Figure S2 **Heatmap based on normalized expression intensity of 101 genes in gene signature between endotoxin and control phosphate buffered saline exposed mice.** Normalized expression intensities been centered to a mean expression of zero across each gene. 2a. Gene signature accurately classifies between endotoxin (LPS) and control (PBS) exposed mice at 8 weeks. 2b. Expression patterns for the 101 genes at 5 days are concordant with those observed at 8 weeks.
(TIF)

Figure S3 **Heatmap based on log_2_ fold change of 101 genes in gene signature in smoke, endotoxin, and smoke plus endotoxin exposed mice as compared to air exposed (control) mice.** Gene signature accurately classifies between these groups.
(TIF)

Table S1 **Annotation of 101 gene signature with gene name and MGI identifier.**
(DOCX)

Table S2 **Pubmed search of genes present in gene signature previously reported to be associated with asthma.**
(DOCX)

Table S3 **Pubmed search of genes present in gene signature previously reported to be associated with COPD.**
(DOCX)
