# Supplementary figures and images for: Integrating Murine Gene Expression Studies to Understand Obstructive Lung Disease Due to Chronic Inhaled Endotoxin
Source: PLoS One. 2013 May 13;8(5):e62910. doi: 10.1371/journal.pone.0062910 (PMC3652821; doi:10.1371/journal.pone.0062910)

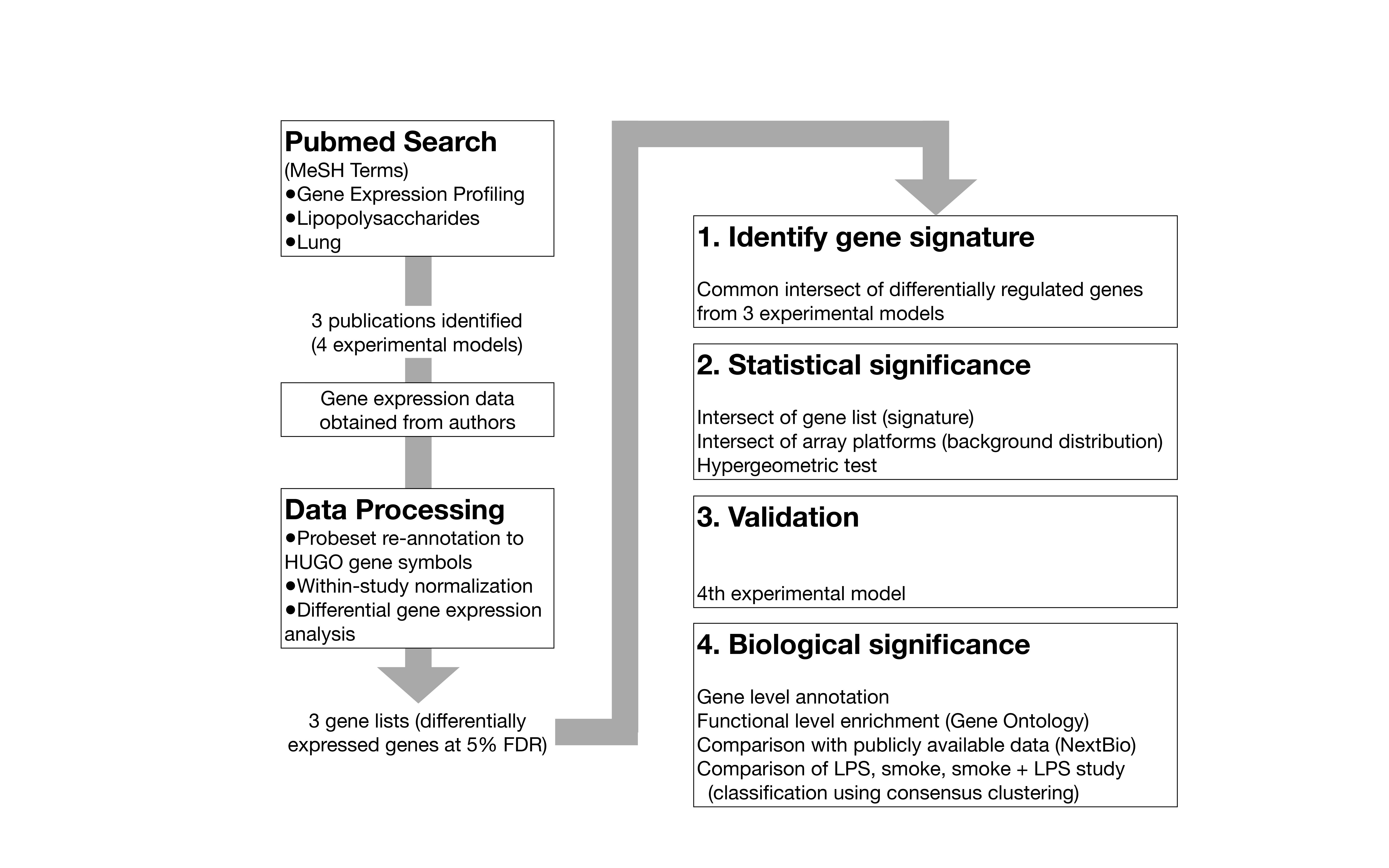

Supplement: Figure S1 — Overview of methods. (TIF) [file pone.0062910.s001.tif]

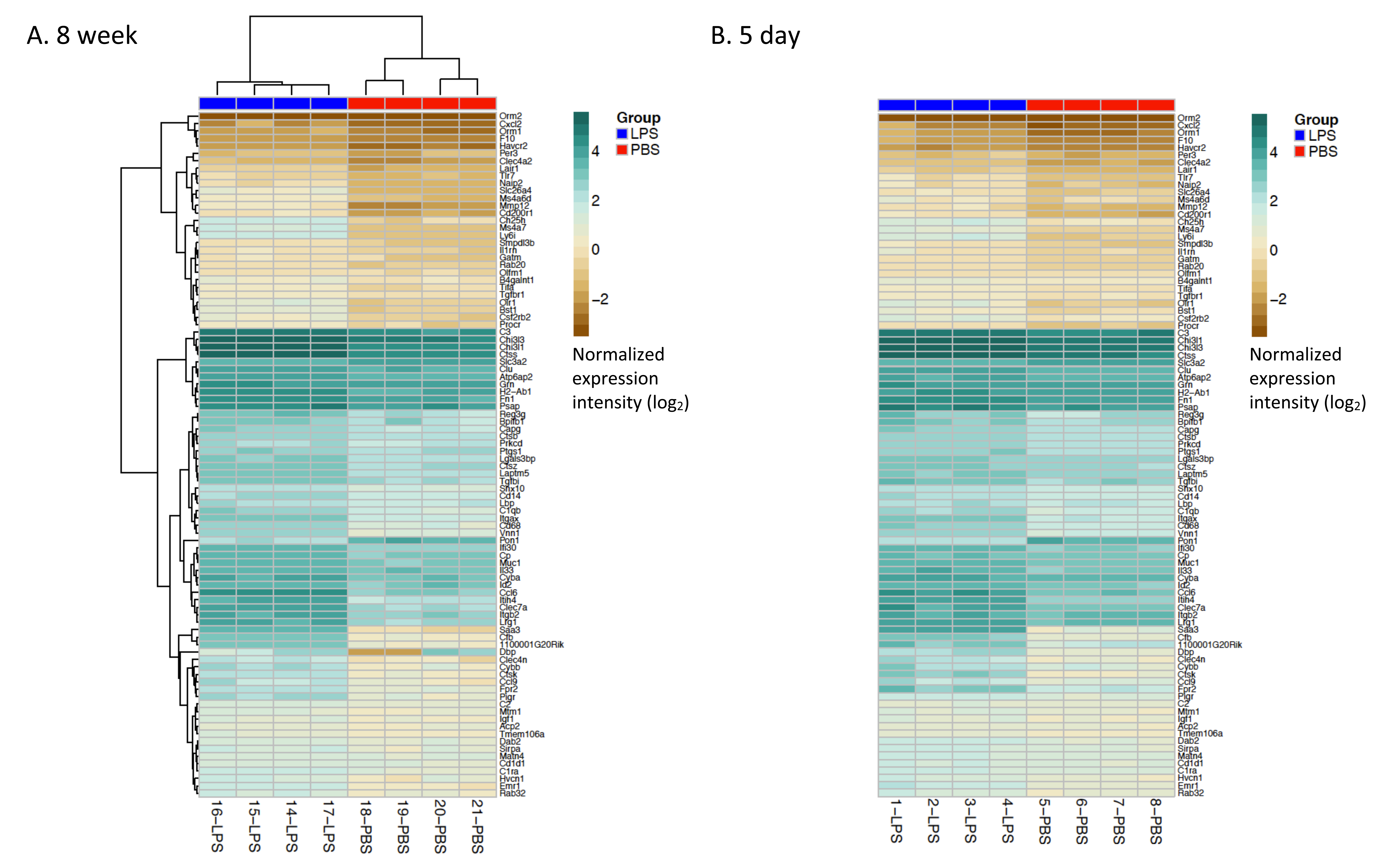

Supplement: Figure S2 — Heatmap based on normalized expression intensity of 101 genes in gene signature between endotoxin and control phosphate buffered saline exposed mice. Normalized expression intensities been centered to a mean expression of zero across each gene. 2a. Gene signature accurately classifies between endotoxin (LPS) and control (PBS) exposed mice at 8 weeks. 2b. Expression patterns for the 101 genes at 5 days are concordant with those observed at 8 weeks. (TIF) [file pone.0062910.s002.tif]
