# Supplementary material for: Integrating Murine Gene Expression Studies to Understand Obstructive Lung Disease Due to Chronic Inhaled Endotoxin
Source: PLoS One. 2013 May 13;8(5):e62910. doi: 10.1371/journal.pone.0062910 (PMC3652821; doi:10.1371/journal.pone.0062910)
Supplement: Table S1 — Annotation of 101 gene signature with gene name and MGI identifier. (DOCX) [file pone.0062910.s004.docx]

**Supplementary Table S1.** Annotation of 101 gene signature with gene name and MGI identifier.

| **MGI symbol** | **MGI Description** | **MGI ID** |
| --- | --- | --- |
| 1100001G20Rik | RIKEN cDNA 1100001G20 gene Gene | MGI:1913357 |
| Acp2 | acid phosphatase 2, lysosomal Gene | MGI:87882 |
| Atp6ap2 | ATPase, H+ transporting, lysosomal accessory protein 2 Gene | MGI:1917745 |
| B4galnt1 | beta-1,4-N-acetyl-galactosaminyl transferase 1 Gene | MGI:1342057 |
| Bcl2a1a | B-cell leukemia/lymphoma 2 related protein A1a Gene | MGI:102687 |
| Bcl2a1b | B-cell leukemia/lymphoma 2 related protein A1b Gene | MGI:1278326 |
| Bcl2a1d | B-cell leukemia/lymphoma 2 related protein A1d Gene | MGI:1278325 |
| Bst1 | bone marrow stromal cell antigen 1 Gene | MGI:105370 |
| C1qb | complement component 1, q subcomponent, beta polypeptide Gene | MGI:88224 |
| C1ra | complement component 1, r subcomponent A Gene | MGI:1355313 |
| C1rb | complement component 1, r subcomponent B Gene | MGI:3779804 |
| C2 | complement component 2 (within H-2S) Gene | MGI:88226 |
| C3 | complement component 3 Gene | MGI:88227 |
| Capg | capping protein (actin filament), gelsolin-like Gene | MGI:1098259 |
| Ccl6 | chemokine (C-C motif) ligand 6 Gene | MGI:98263 |
| Ccl9 | chemokine (C-C motif) ligand 9 Gene | MGI:104533 |
| Cd14 | CD14 antigen Gene | MGI:88318 |
| Cd1d1 | CD1d1 antigen Gene | MGI:107674 |
| Cd200r1 | CD200 receptor 1 Gene | MGI:1889024 |
| Cd68 | CD68 antigen Gene | MGI:88342 |
| Cfb | complement factor B Gene | MGI:105975 |
| Ch25h | cholesterol 25-hydroxylase Gene | MGI:1333869 |
| Chi3l1 | chitinase 3-like 1 Gene | MGI:1340899 |
| Chi3l3 | chitinase 3-like 3 Gene | MGI:1330860 |
| Clec4a2 | C-type lectin domain family 4, member a2 Gene | MGI:1349412 |
| Clec4n | C-type lectin domain family 4, member n Gene | MGI:1861231 |
| Clec7a | C-type lectin domain family 7, member a Gene | MGI:1861431 |
| Clu | clusterin Gene | MGI:88423 |
| Cp | ceruloplasmin Gene | MGI:88476 |
| Csf2rb2 | colony stimulating factor 2 receptor, beta 2, low-affinity (granulocyte-macrophage) Gene | MGI:1339760 |
| Ctsb | cathepsin B Gene | MGI:88561 |
| Ctsk | cathepsin K Gene | MGI:107823 |
| Ctss | cathepsin S Gene | MGI:107341 |
| Ctsz | cathepsin Z Gene | MGI:1891190 |
| Cxcl17 | chemokine (C-X-C motif) ligand 17 Gene | MGI:2387642 |
| Cxcl2 | chemokine (C-X-C motif) ligand 2 Gene | MGI:1340094 |
| Cyba | cytochrome b-245, alpha polypeptide Gene | MGI:1316658 |
| Cybb | cytochrome b-245, beta polypeptide Gene | MGI:88574 |
| Dab2 | disabled homolog 2 (Drosophila) Gene | MGI:109175 |
| Dbp | D site albumin promoter binding protein Gene | MGI:94866 |
| Emr1 | EGF-like module containing, mucin-like, hormone receptor-like sequence 1 Gene | MGI:106912 |
| F10 | coagulation factor X Gene | MGI:103107 |
| Fn1 | fibronectin 1 Gene | MGI:95566 |
| Fpr2 | formyl peptide receptor 2 Gene | MGI:1278319 |
| Gatm | glycine amidinotransferase (L-arginine:glycine amidinotransferase) Gene | MGI:1914342 |
| Grn | granulin Gene | MGI:95832 |
| H2-Ab1 | histocompatibility 2, class II antigen A, beta 1 Gene | MGI:103070 |
| Havcr2 | hepatitis A virus cellular receptor 2 Gene | MGI:2159682 |
| Hvcn1 | hydrogen voltage-gated channel 1 Gene | MGI:1921346 |
| Id2 | inhibitor of DNA binding 2 Gene | MGI:96397 |
| Ifi30 | interferon gamma inducible protein 30 Gene | MGI:2137648 |
| Ifit3 | interferon-induced protein with tetratricopeptide repeats 3 Gene | MGI:1101055 |
| Igf1 | insulin-like growth factor 1 Gene | MGI:96432 |
| Il1rn | interleukin 1 receptor antagonist Gene | MGI:96547 |
| Il33 | interleukin 33 Gene | MGI:1924375 |
| Itgax | integrin alpha X Gene | MGI:96609 |
| Itgb2 | integrin beta 2 Gene | MGI:96611 |
| Itih4 | inter alpha-trypsin inhibitor, heavy chain 4 Gene | MGI:109536 |
| Lair1 | leukocyte-associated Ig-like receptor 1 Gene | MGI:105492 |
| Laptm5 | lysosomal-associated protein transmembrane 5 Gene | MGI:108046 |
| Lbp | lipopolysaccharide binding protein Gene | MGI:1098776 |
| Lgals3bp | lectin, galactoside-binding, soluble, 3 binding protein Gene | MGI:99554 |
| Lrg1 | leucine-rich alpha-2-glycoprotein 1 Gene | MGI:1924155 |
| Ly6i | lymphocyte antigen 6 complex, locus I Gene | MGI:1888480 |
| Matn4 | matrilin 4 Gene | MGI:1328314 |
| Mmp12 | matrix metallopeptidase 12 Gene | MGI:97005 |
| Ms4a6d | membrane-spanning 4-domains, subfamily A, member 6D Gene | MGI:1916024 |
| Ms4a7 | membrane-spanning 4-domains, subfamily A, member 7 Gene | MGI:1918846 |
| Mtm1 | X-linked myotubular myopathy gene 1 Gene | MGI:1099452 |
| Muc1 | mucin 1, transmembrane Gene | MGI:97231 |
| Naip1-rs1 | NLR family, apoptosis inhibitory protein 1, related sequence 1 Pseudogene | MGI:109439 |
| Naip2 | NLR family, apoptosis inhibitory protein 2 Gene | MGI:1298226 |
| Olfm1 | olfactomedin 1 Gene | MGI:1860437 |
| Olr1 | oxidized low density lipoprotein (lectin-like) receptor 1 Gene | MGI:1261434 |
| Orm1 | orosomucoid 1 Gene | MGI:97443 |
| Orm2 | orosomucoid 2 Gene | MGI:97444 |
| Per3 | period homolog 3 (Drosophila) Gene | MGI:1277134 |
| Pigr | polymeric immunoglobulin receptor Gene | MGI:103080 |
| Pon1 | paraoxonase 1 Gene | MGI:103295 |
| Prkcd | protein kinase C, delta Gene | MGI:97598 |
| Procr | protein C receptor, endothelial Gene | MGI:104596 |
| Psap | prosaposin Gene | MGI:97783 |
| Ptgs1 | prostaglandin-endoperoxide synthase 1 Gene | MGI:97797 |
| Rab20 | RAB20, member RAS oncogene family Gene | MGI:102789 |
| Rab32 | RAB32, member RAS oncogene family Gene | MGI:1915094 |
| Reg3g | regenerating islet-derived 3 gamma Gene | MGI:109406 |
| Rmcs2 | response to metastatic cancers 2 Gene | MGI:1890697 |
| Saa3 | serum amyloid A 3 Gene | MGI:98223 |
| Sirpa | signal-regulatory protein alpha Gene | MGI:108563 |
| Slc26a4 | solute carrier family 26, member 4 Gene | MGI:1346029 |
| Slc3a2 | solute carrier family 3 (activators of dibasic and neutral amino acid transport), member 2 Gene | MGI:96955 |
| Slc6a20a | solute carrier family 6 (neurotransmitter transporter), member 20A Gene | MGI:2143217 |
| Smpdl3b | sphingomyelin phosphodiesterase, acid-like 3B Gene | MGI:1916022 |
| Snx10 | sorting nexin 10 Gene | MGI:1919232 |
| Tgfbi | transforming growth factor, beta induced Gene | MGI:99959 |
| Tgfbr1 | transforming growth factor, beta receptor I Gene | MGI:98728 |
| Tifa | TRAF-interacting protein with forkhead-associated domain Gene | MGI:2182965 |
| Tlr7 | toll-like receptor 7 Gene | MGI:2176882 |
| Tmem106a | transmembrane protein 106A Gene | MGI:1922056 |
| U46068 | cDNA sequence U46068 Gene | MGI:2137431 |
| Vnn1 | vanin 1 Gene | MGI:108395 |
