# Supplementary material for: Integrating Murine Gene Expression Studies to Understand Obstructive Lung Disease Due to Chronic Inhaled Endotoxin
Source: PLoS One. 2013 May 13;8(5):e62910. doi: 10.1371/journal.pone.0062910 (PMC3652821; doi:10.1371/journal.pone.0062910)
Supplement: Table S2 — Pubmed search of genes present in gene signature previously reported to be associated with asthma. (DOCX) [file pone.0062910.s005.docx]

**Supplementary Table S2.** Pubmed search of genes present in gene signature previously reported to be associated with asthma.

| symbol | count | pmid | year | journal |
| --- | --- | --- | --- | --- |
| Tgfbi | 0 | 0 |  |  |
| Matn4 | 0 | 0 |  |  |
| Clec7a | 0 | 0 |  |  |
| Olr1 | 1 | 22611474 | 2012 | American journal of translational research |
| Ctsz | 0 | 0 |  |  |
| Mmp12 | 12 | 23075521 | 2012 | International archives of allergy and immunology |
| Mmp12 | 12 | 22837640 | 2012 | Biomarker insights |
| Mmp12 | 12 | 22305682 | 2012 | The Journal of allergy and clinical immunology |
| Mmp12 | 12 | 22216879 | 2012 | Clinical and molecular allergy : CMA |
| Mmp12 | 12 | 20546881 | 2010 | The Journal of allergy and clinical immunology |
| Mmp12 | 12 | 20133923 | 2010 | American journal of respiratory and critical care medicine |
| Mmp12 | 12 | 20018959 | 2009 | The New England journal of medicine |
| Mmp12 | 12 | 19028979 | 2009 | American journal of physiology. Lung cellular and molecular physiology |
| Mmp12 | 12 | 16359550 | 2005 | Respiratory research |
| Mmp12 | 12 | 16166618 | 2005 | American journal of respiratory and critical care medicine |
| Mmp12 | 12 | 15474460 | 2004 | Biochemical and biophysical research communications |
| Mmp12 | 12 | 11893658 | 2002 | Chest |
| Per3 | 0 | 0 |  |  |
| Dab2 | 0 | 0 |  |  |
| Slc3a2 | 0 | 0 |  |  |
| Cyba | 6 | 19459419 | 2009 | TerapevticheskiÄ­ arkhiv |
| Cyba | 6 | 18716406 | 2009 | International archives of allergy and immunology |
| Cyba | 6 | 18672803 | 2008 | Genetika |
| Cyba | 6 | 16608528 | 2006 | Journal of negative results in biomedicine |
| Cyba | 6 | 12594296 | 2003 | Journal of immunology (Baltimore, Md. : 1950) |
| Cyba | 6 | 11940577 | 2002 | The Journal of biological chemistry |
| Fpr2 | 9 | 22410002 | 2012 | Biochemical pharmacology |
| Fpr2 | 9 | 22377711 | 2012 | Journal of human genetics |
| Fpr2 | 9 | 22297737 | 2012 | Archives of pharmacal research |
| Fpr2 | 9 | 21095183 | 2011 | Biochemical pharmacology |
| Fpr2 | 9 | 18583575 | 2008 | American journal of respiratory and critical care medicine |
| Fpr2 | 9 | 17046755 | 2006 | FEBS letters |
| Fpr2 | 9 | 12205450 | 2002 | Nature medicine |
| Fpr2 | 9 | 12172542 | 2002 | Nature medicine |
| Fpr2 | 9 | 12088274 | 2002 | Cellular and molecular life sciences : CMLS |
| Ctsk | 1 | 17311556 | 2007 | Current pharmaceutical design |
| Ctss | 1 | 22170489 | 2012 | The Journal of investigative dermatology |
| Dbp | 12 | 23026126 | 2012 | Water research |
| Dbp | 12 | 22234043 | 2012 | European journal of clinical nutrition |
| Dbp | 12 | 21429484 | 2011 | Environmental research |
| Dbp | 12 | 21370925 | 2011 | Journal of agricultural and food chemistry |
| Dbp | 12 | 19740346 | 2009 | Immunology |
| Dbp | 12 | 18528597 | NA | Revista portuguesa de pneumologia |
| Dbp | 12 | 17969688 | 2007 | Environmental science & technology |
| Dbp | 12 | 16236340 | 2005 | Water research |
| Dbp | 12 | 15103311 | 2004 | Journal of human hypertension |
| Dbp | 12 | 9055100 | 1996 | Indian journal of physiology and pharmacology |
| Dbp | 12 | 1279289 | 1992 | Journal of cardiovascular pharmacology |
| Dbp | 12 | 6507943 | 1984 | Angiology |
| Ctsb | 0 | 0 |  |  |
| Laptm5 | 0 | 0 |  |  |
| Cxcl2 | 30 | 22355409 | 2012 | PloS one |
| Cxcl2 | 30 | 21961642 | 2011 | Journal of environmental science and health |
| Cxcl2 | 30 | 21356202 | 2011 | Chemico-biological interactions |
| Cxcl2 | 30 | 19864593 | 2009 | Journal of immunology (Baltimore, Md. : 1950) |
| Cxcl2 | 30 | 19785013 | 2009 | Stem cells (Dayton, Ohio) |
| Cxcl2 | 30 | 19744573 | 2009 | Pulmonary pharmacology & therapeutics |
| Cxcl2 | 30 | 19671179 | 2009 | Virology journal |
| Cxcl2 | 30 | 19560456 | 2009 | European journal of pharmacology |
| Cxcl2 | 30 | 19050257 | 2008 | Journal of immunology (Baltimore, Md. : 1950) |
| Cxcl2 | 30 | 18787777 | 2008 | Inflammation research |
| Cxcl2 | 30 | 18774390 | 2008 | The Journal of allergy and clinical immunology |
| Cxcl2 | 30 | 18292580 | 2008 | Journal of immunology (Baltimore, Md. : 1950) |
| Cxcl2 | 30 | 18021431 | 2007 | Respiratory research |
| Cxcl2 | 30 | 18007984 | 2007 | Environmental health perspectives |
| Cxcl2 | 30 | 17980417 | 2008 | The Journal of allergy and clinical immunology |
| Cxcl2 | 30 | 17641782 | 2007 | The Journal of clinical investigation |
| Cxcl2 | 30 | 17014439 | 2006 | Clinical and experimental allergy |
| Cxcl2 | 30 | 16929007 | 2006 | Toxicological sciences : an official journal of the Society of Toxicology |
| Cxcl2 | 30 | 16290175 | 2005 | Cytokine |
| Cxcl2 | 30 | 16202497 | 2006 | Toxicology |
| Cxcl2 | 30 | 16122864 | 2005 | Toxicology |
| Cxcl2 | 30 | 15885264 | 2005 | Toxicology and applied pharmacology |
| Cxcl2 | 30 | 15668323 | 2005 | American journal of respiratory cell and molecular biology |
| Cxcl2 | 30 | 15585884 | 2004 | Journal of immunology (Baltimore, Md. : 1950) |
| Cxcl2 | 30 | 14976461 | 2004 | Medical science monitor |
| Cxcl2 | 30 | 12476359 | 2003 | Inhalation toxicology |
| Cxcl2 | 30 | 11766995 | 2001 | Inflammation research |
| Cxcl2 | 30 | 11726396 | 2001 | American journal of respiratory cell and molecular biology |
| Cxcl2 | 30 | 9847020 | 1998 | Veterinary immunology and immunopathology |
| Cxcl2 | 30 | 9490662 | 1998 | American journal of respiratory cell and molecular biology |
| Saa3 | 3 | 22174454 | 2012 | Journal of immunology (Baltimore, Md. : 1950) |
| Saa3 | 3 | 21622869 | 2011 | Journal of immunology (Baltimore, Md. : 1950) |
| Saa3 | 3 | 16973978 | 2006 | American journal of respiratory and critical care medicine |
| Tmem106a | 0 | 0 |  |  |
| Prkcd | 5 | 15536414 | 2004 | The Journal of allergy and clinical immunology |
| Prkcd | 5 | 12759450 | 2003 | Journal of immunology (Baltimore, Md. : 1950) |
| Prkcd | 5 | 12529321 | 2003 | The Journal of biological chemistry |
| Prkcd | 5 | 11748588 | 2001 | Journal of cellular physiology |
| Prkcd | 5 | 9478929 | 1998 | The Journal of biological chemistry |
| Clu | 1 | 10842440 | NA | BoletÃ­n de la AsociaciÃ³n MÃ©dica de Puerto Rico |
| Smpdl3b | 0 | 0 |  |  |
| Lair1 | 0 | 0 |  |  |
| Itih4 | 0 | 0 |  |  |
| Grn | 0 | 0 |  |  |
| Tgfbr1 | 0 | 0 |  |  |
| Lrg1 | 0 | 0 |  |  |
| Cd1d1 | 2 | 20083656 | 2010 | Journal of immunology (Baltimore, Md. : 1950) |
| Cd1d1 | 2 | 9927517 | 1999 | The Journal of experimental medicine |
| C3 | 143 | 23113211 | 2012 | Iranian journal of public health |
| C3 | 143 | 22997700 | NA | Bioorganicheskaia khimiia |
| C3 | 143 | 22734375 | 2012 | Zhongguo zhen jiu = Chinese acupuncture & moxibustion |
| C3 | 143 | 22567103 | 2012 | PloS one |
| C3 | 143 | 22372350 | 2012 | Clinical laboratory |
| C3 | 143 | 22367138 | 2012 | Medical science monitor |
| C3 | 143 | 22361510 | 2012 | Allergology international |
| C3 | 143 | 22246175 | 2012 | American journal of respiratory and critical care medicine |
| C3 | 143 | 22211906 | 2012 | Clinical and experimental allergy |
| C3 | 143 | 22149063 | 2012 | Journal of aerosol medicine and pulmonary drug delivery |
| C3 | 143 | 22065014 | NA | Jornal de pediatria |
| C3 | 143 | 21846943 | 2011 | Disease markers |
| C3 | 143 | 21801245 | 2012 | Clinical and experimental allergy |
| C3 | 143 | 21620804 | 2011 | Biochemical pharmacology |
| C3 | 143 | 21480785 | 2011 | Journal of alternative and complementary medicine (New York, N.Y.) |
| C3 | 143 | 21462136 | 2011 | Chinese journal of medical genetics |
| C3 | 143 | 21210563 | 2004 |  |
| C3 | 143 | 20696559 | 2010 | Phytomedicine |
| C3 | 143 | 20600518 | 2010 | Food and chemical toxicology |
| C3 | 143 | 20589464 | 2010 | Indian journal of pediatrics |
| C3 | 143 | 20538303 | 2010 | Journal of the neurological sciences |
| C3 | 143 | 20511342 | 2010 | American journal of physiology. Lung cellular and molecular physiology |
| C3 | 143 | 20402389 | 2010 | Expert review of clinical immunology |
| C3 | 143 | 20395963 | 2010 | Journal of human genetics |
| C3 | 143 | 20029843 | 2010 | Proteomics |
| C3 | 143 | 19684087 | 2009 | Journal of immunology (Baltimore, Md. : 1950) |
| C3 | 143 | 22557323 | 2009 | Ancient science of life |
| C3 | 143 | 18566738 | 2008 | Journal of human genetics |
| C3 | 143 | 18424754 | 2008 | Journal of immunology (Baltimore, Md. : 1950) |
| C3 | 143 | 19823661 | 2008 | Indian journal of orthopaedics |
| C3 | 143 | 17418355 | NA | Neurophysiologie clinique = Clinical neurophysiology |
| C3 | 143 | 17082579 | 2006 | Journal of immunology (Baltimore, Md. : 1950) |
| C3 | 143 | 17002917 | 2006 | Translational research : the journal of laboratory and clinical medicine |
| C3 | 143 | 16913666 | 2006 | Rinsho byori. The Japanese journal of clinical pathology |
| C3 | 143 | 16879240 | 2006 | Clinical and experimental immunology |
| C3 | 143 | 16858009 | 2006 | American journal of respiratory cell and molecular biology |
| C3 | 143 | 16574942 | 2006 | American journal of respiratory cell and molecular biology |
| C3 | 143 | 16439722 | 2006 | American journal of respiratory and critical care medicine |
| C3 | 143 | 16355111 | 2006 | Genes and immunity |
| C3 | 143 | 16312927 | 2005 | Zhongguo zhen jiu = Chinese acupuncture & moxibustion |
| C3 | 143 | 16293803 | 2006 | American journal of respiratory and critical care medicine |
| C3 | 143 | 16186675 | 2005 | Indian journal of pediatrics |
| C3 | 143 | 16113417 | 2004 | Proceedings of the American Thoracic Society |
| C3 | 143 | 16091207 | 2005 | Current allergy and asthma reports |
| C3 | 143 | 15655303 | 2004 | Journal of smooth muscle research |
| C3 | 143 | 15638941 | 2005 | Respiratory research |
| C3 | 143 | 15338393 | 2004 | Pediatric nephrology (Berlin, Germany) |
| C3 | 143 | 15278436 | 2004 | Human genetics |
| C3 | 143 | 12097289 | 2002 | Cancer research |
| C3 | 143 | 12096683 | 2002 | Central European journal of public health |
| C3 | 143 | 11980156 | 2002 | Klinicheskaia meditsina |
| C3 | 143 | 11979168 | 2002 | Spine |
| C3 | 143 | 11591733 | 2001 | Journal of immunology (Baltimore, Md. : 1950) |
| C3 | 143 | 11510804 | 2001 | The European respiratory journal |
| C3 | 143 | 11477893 | 1998 | Chinese journal of tuberculosis and respiratory diseases |
| C3 | 143 | 11189891 | 2000 | Handchirurgie, Mikrochirurgie, plastische Chirurgie |
| C3 | 143 | 10826222 | 2000 | Hukuoka acta medica |
| C3 | 143 | 10660972 | NA | Romanian journal of internal medicine |
| C3 | 143 | 10553582 | 1999 | Nihon yakurigaku zasshi. Folia pharmacologica Japonica |
| C3 | 143 | 10594541 | 1999 | Clinical and experimental allergy |
| C3 | 143 | 9538632 | 1998 | The Korean journal of internal medicine |
| C3 | 143 | 9440942 | 1997 | Meditsina truda i promyshlennaia ekologiia |
| C3 | 143 | 8843003 | 1996 | Journal of Korean medical science |
| C3 | 143 | 8563490 | 1996 | International archives of allergy and immunology |
| C3 | 143 | 8520733 | 1995 | American journal of respiratory and critical care medicine |
| C3 | 143 | 7752082 | 1995 | The Journal of pharmacology and experimental therapeutics |
| C3 | 143 | 7950448 | NA | Pneumoftiziologia] |
| C3 | 143 | 8443468 | 1993 | International archives of allergy and immunology |
| C3 | 143 | 8428161 | 1993 | International archives of allergy and immunology |
| C3 | 143 | 1558328 | 1992 | Annals of allergy |
| C3 | 143 | 1548406 | 1992 | Journal of immunological methods |
| C3 | 143 | 1476040 | 1992 | Acta dermato-venereologica. Supplementum |
| C3 | 143 | 1345603 | 1992 | Bulletin of the Institute of Maritime and Tropical Medicine in Gdynia |
| C3 | 143 | 1659436 | 1991 | British journal of clinical pharmacology |
| C3 | 143 | 1772350 | 1991 | ArerugÄ« = [Allergy] |
| C3 | 143 | 1953911 | 1991 | The European respiratory journal. Supplement |
| C3 | 143 | 1773456 | 1991 | Chinese journal of modern developments in traditional medicine |
| C3 | 143 | 1669567 | 1991 | Journal of investigational allergology & clinical immunology |
| C3 | 143 | 1809688 | 1991 | International archives of allergy and applied immunology |
| C3 | 143 | 2221489 | 1990 | Annals of allergy |
| C3 | 143 | 2129476 | NA | Archivos de investigaciÃ³n mÃ©dica |
| C3 | 143 | 2802267 | 1989 | Annals of allergy |
| C3 | 143 | 2721280 | 1989 | Chest |
| C3 | 143 | 2526632 | 1989 | Asian Pacific journal of allergy and immunology |
| C3 | 143 | 3272988 | NA | Zhonghua Minguo xiao er ke yi xue hui za zhi [Journal] |
| C3 | 143 | 3258826 | 1988 | The European respiratory journal |
| C3 | 143 | 3545262 | 1987 | Archives of otolaryngology--head & neck surgery |
| C3 | 143 | 3506436 | 1987 | Bulletin of the Institute of Maritime and Tropical Medicine in Gdynia |
| C3 | 143 | 3491553 | 1986 | Annals of allergy |
| C3 | 143 | 3717764 | 1986 | The American review of respiratory disease |
| C3 | 143 | 2951829 | 1986 | Respiration; international review of thoracic diseases |
| C3 | 143 | 4050221 | 1985 | Zhurnal mikrobiologii, epidemiologii, i immunobiologii |
| C3 | 143 | 4033033 | 1985 | Klinicheskaia meditsina |
| C3 | 143 | 3893231 | 1985 | Annals of allergy |
| C3 | 143 | 4029967 | 1985 | Human heredity |
| C3 | 143 | 6442583 | 1984 | Asian Pacific journal of allergy and immunology |
| C3 | 143 | 6210004 | 1984 | The American review of respiratory disease |
| C3 | 143 | 6528951 | NA | AlergÃ­a |
| C3 | 143 | 6085153 | 1984 | Pneumonologia polska |
| C3 | 143 | 6499053 | 1984 | CeskoslovenskÃ¡ pediatrie |
| C3 | 143 | 6465480 | 1984 | Allergy |
| C3 | 143 | 6429229 | 1984 | The Journal of allergy and clinical immunology |
| C3 | 143 | 6201833 | 1984 | Pneumonologia polska |
| C3 | 143 | 6719352 | 1984 | TerapevticheskiÄ­ arkhiv |
| C3 | 143 | 6627619 | 1983 | Clinical allergy |
| C3 | 143 | 6544421 | NA | La Pediatria medica e chirurgica |
| C3 | 143 | 6342165 | 1983 | South African medical journal |
| C3 | 143 | 6878777 | 1983 | Revista clÃ­nica espaÃ±ola |
| C3 | 143 | 6194517 | 1983 | Pneumonologia polska |
| C3 | 143 | 6831684 | 1983 | Clinical allergy |
| C3 | 143 | 6924864 | 1982 | Clinical allergy |
| C3 | 143 | 6919393 | NA | Allergologia et immunopathologia |
| C3 | 143 | 6797794 | 1982 | Chest |
| C3 | 143 | 6975678 | 1981 | Clinical and experimental immunology |
| C3 | 143 | 6781385 | 1981 | Annals of allergy |
| C3 | 143 | 6975491 | 1981 | Respiration; international review of thoracic diseases |
| C3 | 143 | 7444701 | 1980 | South African medical journal |
| C3 | 143 | 6768786 | 1980 | The Journal of allergy and clinical immunology |
| C3 | 143 | 539520 | NA | Allergologia et immunopathologia |
| C3 | 143 | 382104 | 1979 | La Nouvelle presse mÃ©dicale |
| C3 | 143 | 156917 | 1979 | Praxis und Klinik der Pneumologie |
| C3 | 143 | 445782 | 1979 | Clinical allergy |
| C3 | 143 | 115071 | NA | Revue franÃ§aise des maladies respiratoires |
| C3 | 143 | 162027 | 1979 | Allergie und Immunologie |
| C3 | 143 | 752256 | NA | Allergologia et immunopathologia |
| C3 | 143 | 373930 | 1978 | Clinical and experimental immunology |
| C3 | 143 | 709794 | 1978 | Clinical allergy |
| C3 | 143 | 686507 | 1978 | Annals of allergy |
| C3 | 143 | 677979 | 1978 | Archives of internal medicine |
| C3 | 143 | 308809 | 1978 | British journal of diseases of the chest |
| C3 | 143 | 309715 | NA | Allergologia et immunopathologia |
| C3 | 143 | 652026 | 1978 | Mycopathologia |
| C3 | 143 | 627043 | 1978 | Clinical allergy |
| C3 | 143 | 846785 | 1977 | Pediatric research |
| C3 | 143 | 324512 | 1977 | The British journal of dermatology |
| C3 | 143 | 67566 | 1977 | Nature |
| C3 | 143 | 872357 | 1977 | Clinical allergy |
| C3 | 143 | 988767 | 1976 | The American review of respiratory disease |
| C3 | 143 | 968799 | 1976 | Thorax |
| C3 | 143 | 56632 | 1976 | Lancet |
| C3 | 143 | 58741 | 1976 | Clinical allergy |
| C3 | 143 | 1099944 | 1975 | Annals of allergy |
| C3 | 143 | 1100092 | 1975 | The British journal of dermatology |
| LOC100048759 | 0 | 0 |  |  |
| Fn1 | 1 | 19710636 | 2010 | Mucosal immunology |
| Emr1 | 1 | 20625511 | 2010 | PloS one |
| Chi3l3 | 7 | 22014099 | 2011 | BMC immunology |
| Chi3l3 | 7 | 21530272 | 2011 | Bioorganic & medicinal chemistry |
| Chi3l3 | 7 | 21469115 | 2011 | European journal of immunology |
| Chi3l3 | 7 | 18758056 | 2008 | Biological & pharmaceutical bulletin |
| Chi3l3 | 7 | 18087596 | 2007 | Environmental health perspectives |
| Chi3l3 | 7 | 17082650 | 2006 | Journal of immunology (Baltimore, Md. : 1950) |
| Chi3l3 | 7 | 11553626 | 2001 | The Journal of biological chemistry |
| Acp2 | 0 | 0 |  |  |
| H2-Ab1 | 0 | 0 |  |  |
| Havcr2 | 18 | 21623966 | 2011 | Clinical and experimental allergy |
| Havcr2 | 18 | 21575348 | 2011 | Chinese journal of contemporary pediatrics |
| Havcr2 | 18 | 21470319 | 2011 | Clinical and experimental allergy |
| Havcr2 | 18 | 20536563 | 2010 | Immunological reviews |
| Havcr2 | 18 | 20083673 | 2010 | Journal of immunology (Baltimore, Md. : 1950) |
| Havcr2 | 18 | 19905911 | 2009 | The Journal of asthma |
| Havcr2 | 18 | 19566956 | 2009 | BMC medical genetics |
| Havcr2 | 18 | 19494522 | 2009 | International archives of allergy and immunology |
| Havcr2 | 18 | 18785518 | 2008 | Chinese journal of tuberculosis and respiratory diseases |
| Havcr2 | 18 | 18727494 | 2008 | Current topics in microbiology and immunology |
| Havcr2 | 18 | 16456792 | 2006 | Chinese journal of medical genetics |
| Havcr2 | 18 | 16002337 | 2005 | Trends in molecular medicine |
| Havcr2 | 18 | 15867855 | 2005 | The Journal of allergy and clinical immunology |
| Havcr2 | 18 | 15603868 | 2004 | Human immunology |
| Havcr2 | 18 | 15272240 | 2004 | Experimental & molecular medicine |
| Havcr2 | 18 | 14999428 | 2004 | Springer seminars in immunopathology |
| Havcr2 | 18 | 14508299 | 2003 | Current opinion in pediatrics |
| Havcr2 | 18 | 11725301 | 2001 | Nature immunology |
| Cfb | 0 | 0 |  |  |
| C2 | 43 | 22826050 | 2013 | Advances in experimental medicine and biology |
| C2 | 43 | 22458856 | 2012 | Journal of environmental science and health |
| C2 | 43 | 22142423 | 2012 | Journal of medicinal chemistry |
| C2 | 43 | 22094623 | 2012 | Respiration; international review of thoracic diseases |
| C2 | 43 | 21889615 | 2011 | Journal of biomedical informatics |
| C2 | 43 | 20600518 | 2010 | Food and chemical toxicology |
| C2 | 43 | 20368027 | 2010 | Chinese journal of tuberculosis and respiratory diseases |
| C2 | 43 | 19651244 | 2009 | Respiratory physiology & neurobiology |
| C2 | 43 | 18842290 | 2008 | The Journal of allergy and clinical immunology |
| C2 | 43 | 17379851 | 2007 | American journal of respiratory and critical care medicine |
| C2 | 43 | 17305324 | 2007 | Journal of medicinal chemistry |
| C2 | 43 | 16843616 | 2006 | Medical hypotheses |
| C2 | 43 | 16840383 | 2006 | Chest |
| C2 | 43 | 16061704 | 2005 | Thorax |
| C2 | 43 | 15853649 | 2005 | Current protein & peptide science |
| C2 | 43 | 15805998 | 2005 | The Journal of allergy and clinical immunology |
| C2 | 43 | 15135092 | 2004 | Journal of chromatography. |
| C2 | 43 | 14642800 | 2003 | Clinical therapeutics |
| C2 | 43 | 12872723 | 2003 | Nihon Jibiinkoka Gakkai kaiho |
| C2 | 43 | 12693800 | 2003 | Respiratory medicine |
| C2 | 43 | 12495964 | 2003 | Archives of disease in childhood |
| C2 | 43 | 12184862 | 2002 | Journal of aerosol medicine |
| C2 | 43 | 12153960 | 2002 | American journal of respiratory and critical care medicine |
| C2 | 43 | 11979168 | 2002 | Spine |
| C2 | 43 | 11964752 | 2002 | Current opinion in allergy and clinical immunology |
| C2 | 43 | 11477893 | 1998 | Chinese journal of tuberculosis and respiratory diseases |
| C2 | 43 | 11049743 | 2000 | Protein expression and purification |
| C2 | 43 | 11042185 | 2001 | The Journal of biological chemistry |
| C2 | 43 | 10780759 | 2000 | The European respiratory journal |
| C2 | 43 | 10350223 | 1999 | The Journal of asthma |
| C2 | 43 | 10319815 | 1999 | Cell |
| C2 | 43 | 9751271 | 1998 | Cancer letters |
| C2 | 43 | 9592812 | 1998 | No shinkei geka. Neurological surgery |
| C2 | 43 | 8613071 | 1996 | Gastroenterology |
| C2 | 43 | 1444832 | 1992 | ArerugÄ« = [Allergy] |
| C2 | 43 | 1348480 | 1992 | The European respiratory journal |
| C2 | 43 | 2221489 | 1990 | Annals of allergy |
| C2 | 43 | 3041356 | 1988 | The Pediatric infectious disease journal |
| C2 | 43 | 3773900 | 1986 | Monographs in allergy |
| C2 | 43 | 6788213 | 1981 | British medical journal (Clinical research ed.) |
| C2 | 43 | 7438416 | 1980 | Clinical allergy |
| C2 | 43 | 710825 | 1978 | Gastroenterologia Japonica |
| C2 | 43 | 62112 | 1976 | Lancet |
| Cp | 105 | 23091170 | 2012 | Toxicological sciences |
| Cp | 105 | 22823210 | 2012 | Respiratory research |
| Cp | 105 | 22685462 | 2012 | International journal of otolaryngology |
| Cp | 105 | 22678519 | 2012 | Indian journal of pediatrics |
| Cp | 105 | 22545149 | 2012 | PloS one |
| Cp | 105 | 22329284 | 2011 | CasopÃ­s lÃ©karÌ†Å¯ cÌ†eskÃ½ch |
| Cp | 105 | 22300433 | 2012 | Pediatric allergy and immunology |
| Cp | 105 | 21695198 | 2011 | PloS one |
| Cp | 105 | 21573487 | 2011 | International journal of molecular medicine |
| Cp | 105 | 21439045 | 2011 | Biomedical engineering online |
| Cp | 105 | 21157643 | 2010 | Current opinion in investigational drugs (London, England : 2000) |
| Cp | 105 | 20190433 | 2010 | Chemical & pharmaceutical bulletin |
| Cp | 105 | 19998041 | 2010 | Lung |
| Cp | 105 | 19905928 | 2009 | The Journal of asthma |
| Cp | 105 | 19768975 | NA | Revista alergia Mexico (Tecamachalco, Puebla, Mexico : 1993) |
| Cp | 105 | 20873055 | 2009 | Revista alergia Mexico (Tecamachalco, Puebla, Mexico : 1993) |
| Cp | 105 | 19058490 | NA | Revista alergia Mexico (Tecamachalco, Puebla, Mexico : 1993) |
| Cp | 105 | 19052510 | 2008 | ArerugÄ« = [Allergy] |
| Cp | 105 | 19010996 | 2009 | The European respiratory journal |
| Cp | 105 | 18693537 | NA | Revista alergia Mexico (Tecamachalco, Puebla, Mexico : 1993) |
| Cp | 105 | 18601933 | 2008 | Life sciences |
| Cp | 105 | 18397914 | 2008 | American journal of epidemiology |
| Cp | 105 | 18266975 | 2008 | Journal of cellular and molecular medicine |
| Cp | 105 | 18259994 | NA | The Journal of asthma |
| Cp | 105 | 18242596 | 2008 | European journal of pharmacology |
| Cp | 105 | 18188083 | 2008 | Journal of occupational and environmental medicine |
| Cp | 105 | 19462122 | 2007 | Revista brasileira de anestesiologia |
| Cp | 105 | 17384874 | 2007 | Singapore medical journal |
| Cp | 105 | 17287299 | 2007 | Thorax |
| Cp | 105 | 17251674 | 2007 | Circulation journal |
| Cp | 105 | 17124849 | 2006 | Equine veterinary journal |
| Cp | 105 | 17121872 | 2007 | Thorax |
| Cp | 105 | 16801164 | 2006 | Acta paediatrica (Oslo, Norway : 1992). Supplement |
| Cp | 105 | 16624877 | 2006 | Proceedings of the National Academy of Sciences of the United States of America |
| Cp | 105 | 16613702 | 2006 | Chinese journal of contemporary pediatrics |
| Cp | 105 | 16575135 | 2006 | Georgian medical news |
| Cp | 105 | 16395708 | 2006 | International journal of cancer. Journal international du cancer |
| Cp | 105 | 16158778 | NA | Revista alergia Mexico (Tecamachalco, Puebla, Mexico : 1993) |
| Cp | 105 | 15946835 | 2006 | Respiratory medicine |
| Cp | 105 | 15787872 | 2005 | Pediatric allergy and immunology |
| Cp | 105 | 15753914 | 2005 | The Journal of allergy and clinical immunology |
| Cp | 105 | 15659480 | 2005 | BMJ (Clinical research ed.) |
| Cp | 105 | 15598725 | 2005 | Journal of epidemiology and community health |
| Cp | 105 | 15575487 | 2004 | TerapevticheskiÄ­ arkhiv |
| Cp | 105 | 15565789 | 2004 | Seminars in perinatology |
| Cp | 105 | 15482516 | 2004 | Pediatric allergy and immunology |
| Cp | 105 | 15477001 | 2004 | Patient education and counseling |
| Cp | 105 | 15286255 | 2004 | Pediatrics |
| Cp | 105 | 15241925 | NA | Zeitschrift fÃ¼r Naturforschung. C, Journal of biosciences |
| Cp | 105 | 15066221 | 2004 | Acta pharmacologica Sinica |
| Cp | 105 | 14968984 | NA | Revista alergia Mexico (Tecamachalco, Puebla, Mexico : 1993) |
| Cp | 105 | 14964011 | 2003 | SantÃ© publique (Vandoeuvre-lÃ¨s-Nancy, France) |
| Cp | 105 | 12940106 | NA | Revista alergia Mexico (Tecamachalco, Puebla, Mexico : 1993) |
| Cp | 105 | 12822543 | NA | Revista alergia Mexico (Tecamachalco, Puebla, Mexico : 1993) |
| Cp | 105 | 12822542 | NA | Revista alergia Mexico (Tecamachalco, Puebla, Mexico : 1993) |
| Cp | 105 | 12530578 | 2002 | Pathology, research and practice |
| Cp | 105 | 12441327 | 2002 | Journal of the National Cancer Institute |
| Cp | 105 | 12371533 | 2002 | Journal of investigational allergology & clinical immunology |
| Cp | 105 | 12205810 | 2002 | Archives de pÃ©diatrie |
| Cp | 105 | 12011732 | 2002 | Revue d'Ã©pidÃ©miologie et de santÃ© publique |
| Cp | 105 | 11961081 | 2002 | The Journal of pharmacology and experimental therapeutics |
| Cp | 105 | 11862756 | 2002 | Nihon yakurigaku zasshi. Folia pharmacologica Japonica |
| Cp | 105 | 11721274 | 2001 | Annals of the Academy of Medicine, Singapore |
| Cp | 105 | 11668926 | 2001 | Australian health review |
| Cp | 105 | 11602515 | 2001 | Drug metabolism and disposition: the biological fate of chemicals |
| Cp | 105 | 11426850 | 2001 | European journal of pharmacology |
| Cp | 105 | 11401872 | 2001 | American journal of respiratory and critical care medicine |
| Cp | 105 | 11273793 | 2001 | Pulmonary pharmacology & therapeutics |
| Cp | 105 | 11171871 | 2001 | International journal of epidemiology |
| Cp | 105 | 11003986 | 2000 | American journal of physiology. Regulatory, integrative and comparative physiology |
| Cp | 105 | 10988132 | 2000 | American journal of respiratory and critical care medicine |
| Cp | 105 | 10903239 | 2000 | American journal of respiratory and critical care medicine |
| Cp | 105 | 10891017 | 2000 | Archives of pediatrics & adolescent medicine |
| Cp | 105 | 10710030 | 2000 | Archives of pediatrics & adolescent medicine |
| Cp | 105 | 10611439 | 1999 | European journal of pharmacology |
| Cp | 105 | 10468306 | 1999 | British journal of cancer |
| Cp | 105 | 10235630 | 1999 | Regulatory peptides |
| Cp | 105 | 9927373 | 1999 | American journal of respiratory and critical care medicine |
| Cp | 105 | 9723564 | 1998 | Annals of allergy, asthma & immunology |
| Cp | 105 | 9400681 | 1997 | Respirology (Carlton, Vic.) |
| Cp | 105 | 9353399 | 1997 | The Journal of pharmacology and experimental therapeutics |
| Cp | 105 | 11498865 | 1997 | Acta pharmaceutica Sinica |
| Cp | 105 | 9234081 | 1997 | Journal of autonomic pharmacology |
| Cp | 105 | 8836335 | 1996 | Allergy |
| Cp | 105 | 8567958 | 1996 | The Journal of clinical investigation |
| Cp | 105 | 7663799 | 1995 | American journal of respiratory and critical care medicine |
| Cp | 105 | 8846432 | 1995 | Canadian journal of physiology and pharmacology |
| Cp | 105 | 7735167 | 1995 | Nuclear medicine and biology |
| Cp | 105 | 8121098 | 1993 | Nihon KyÅbu Shikkan Gakkai zasshi |
| Cp | 105 | 7693493 | 1993 | European journal of pharmacology |
| Cp | 105 | 7692490 | 1993 | Regulatory peptides |
| Cp | 105 | 7902346 | 1993 | The Journal of asthma |
| Cp | 105 | 1525326 | 1992 | Cancer causes & control : CCC |
| Cp | 105 | 1990954 | 1991 | The American review of respiratory disease |
| Cp | 105 | 2251634 | 1990 | South African medical journal |
| Cp | 105 | 2247790 | 1990 | South African medical journal |
| Cp | 105 | 2339312 | 1990 | South African medical journal |
| Cp | 105 | 2896105 | 1988 | Chest |
| Cp | 105 | 3340935 | 1988 | South African medical journal |
| Cp | 105 | 2443279 | 1987 | Clinica chimica acta |
| Cp | 105 | 6233230 | 1984 | International journal of immunopharmacology |
| Cp | 105 | 7065516 | 1982 | The American review of respiratory disease |
| Cp | 105 | 7129659 | 1982 | International archives of allergy and applied immunology |
| Cp | 105 | 13094 | 1977 | The Journal of allergy and clinical immunology |
| Cp | 105 | 993478 | 1976 | The Journal of allergy and clinical immunology |
| Ly6i | 0 | 0 |  |  |
| Pigr | 1 | 22240167 | 2012 | Biochimica et biophysica acta |
| C1qb | 0 | 0 |  |  |
| Bst1 | 0 | 0 |  |  |
| Muc1 | 6 | 21605280 | 2011 | Pediatrics international : official journal of the Japan Pediatric Society |
| Muc1 | 6 | 20348949 | 2010 | Oncogene |
| Muc1 | 6 | 16990615 | 2007 | American journal of respiratory cell and molecular biology |
| Muc1 | 6 | 16630149 | 2006 | Clinical and experimental allergy |
| Muc1 | 6 | 11802251 | 2002 | Pediatric pulmonology |
| Muc1 | 6 | 11062147 | 2000 | American journal of respiratory cell and molecular biology |
| Lgals3bp | 1 | 15562889 | 2004 | Annals of allergy, asthma & immunology |
| Hvcn1 | 1 | 19958596 | NA | American journal of rhinology & allergy |
| Slc6a20a | 0 | 0 |  |  |
| Orm1 | 10 | 23096927 | 2012 | Genetics and molecular research : GMR |
| Orm1 | 10 | 22986918 | 2012 | The pharmacogenomics journal |
| Orm1 | 10 | 22694930 | 2012 | The Journal of allergy and clinical immunology |
| Orm1 | 10 | 22535525 | 2012 | Molecular biology of the cell |
| Orm1 | 10 | 22271045 | 2012 | Human genetics |
| Orm1 | 10 | 22069270 | 2011 | Diabetes/metabolism research and reviews |
| Orm1 | 10 | 22017802 | 2012 | International journal of immunogenetics |
| Orm1 | 10 | 20182505 | 2010 | Nature |
| Orm1 | 10 | 19133921 | 2009 | Allergy |
| Orm1 | 10 | 18155279 | 2008 | The Journal of allergy and clinical immunology |
| Orm2 | 2 | 22535525 | 2012 | Molecular biology of the cell |
| Orm2 | 2 | 20182505 | 2010 | Nature |
| Bcl2a1d | 0 | 0 |  |  |
| Bcl2a1a | 0 | 0 |  |  |
| Bcl2a1b | 0 | 0 |  |  |
| Csf2rb2 | 1 | 21841801 | 2011 | Nature |
| Tifa | 0 | 0 |  |  |
| Itgax | 58 | 22585735 | 2012 | The Journal of experimental medicine |
| Itgax | 58 | 22388091 | 2012 | Nature medicine |
| Itgax | 58 | 22110701 | 2011 | PloS one |
| Itgax | 58 | 21985360 | 2011 | Clinical and experimental immunology |
| Itgax | 58 | 21646790 | 2011 | International archives of allergy and immunology |
| Itgax | 58 | 21634009 | 2011 | EMBO molecular medicine |
| Itgax | 58 | 21538995 | 2011 | EMBO molecular medicine |
| Itgax | 58 | 21477339 | 2011 | Respiratory research |
| Itgax | 58 | 21402950 | 2011 | Proceedings of the National Academy of Sciences of the United States of America |
| Itgax | 58 | 21274737 | 2011 | Inflammation research |
| Itgax | 58 | 21268008 | 2011 | European journal of immunology |
| Itgax | 58 | 21231886 | 2011 | Immunopharmacology and immunotoxicology |
| Itgax | 58 | 21135031 | 2011 | International immunology |
| Itgax | 58 | 20819092 | 2010 | Clinical and experimental immunology |
| Itgax | 58 | 20659336 | 2010 | Respiratory research |
| Itgax | 58 | 20622891 | 2010 | Cellular & molecular immunology |
| Itgax | 58 | 20581095 | 2011 | American journal of respiratory cell and molecular biology |
| Itgax | 58 | 20375632 | 2010 | Journal of innate immunity |
| Itgax | 58 | 20351460 | 2010 | Journal of infection in developing countries |
| Itgax | 58 | 20214669 | 2010 | Clinical and experimental allergy |
| Itgax | 58 | 20194813 | 2010 | American journal of respiratory and critical care medicine |
| Itgax | 58 | 20179765 | 2010 | PloS one |
| Itgax | 58 | 20118218 | 2011 | American journal of respiratory cell and molecular biology |
| Itgax | 58 | 20085598 | 2009 | Clinical and experimental allergy |
| Itgax | 58 | 20016195 | 2010 | International archives of allergy and immunology |
| Itgax | 58 | 19933379 | 2010 | American journal of respiratory cell and molecular biology |
| Itgax | 58 | 19901344 | 2010 | American journal of respiratory cell and molecular biology |
| Itgax | 58 | 19877020 | 2009 | European journal of immunology |
| Itgax | 58 | 19828636 | 2009 | Journal of immunology (Baltimore, Md. : 1950) |
| Itgax | 58 | 19628980 | 2009 | Allergology international |
| Itgax | 58 | 19553159 | 2009 | Clinical immunology (Orlando, Fla.) |
| Itgax | 58 | 19494498 | 2009 | International archives of allergy and immunology |
| Itgax | 58 | 19464382 | 2009 | Pulmonary pharmacology & therapeutics |
| Itgax | 58 | 19448155 | 2010 | American journal of respiratory cell and molecular biology |
| Itgax | 58 | 19155511 | 2009 | Journal of immunology (Baltimore, Md. : 1950) |
| Itgax | 58 | 18835962 | 2009 | Thorax |
| Itgax | 58 | 18594149 | 2008 | International archives of allergy and immunology |
| Itgax | 58 | 18498542 | 2008 | Clinical and experimental allergy |
| Itgax | 58 | 18209085 | 2008 | Journal of immunology (Baltimore, Md. : 1950) |
| Itgax | 58 | 17977814 | 2007 | International immunology |
| Itgax | 58 | 17512567 | 2007 | Toxicology and applied pharmacology |
| Itgax | 58 | 17506035 | 2007 | European journal of immunology |
| Itgax | 58 | 17460444 | 2007 | Allergology international |
| Itgax | 58 | 17210044 | 2007 | Clinical and experimental allergy |
| Itgax | 58 | 16455972 | 2006 | Journal of immunology (Baltimore, Md. : 1950) |
| Itgax | 58 | 16424176 | 2006 | Journal of immunology (Baltimore, Md. : 1950) |
| Itgax | 58 | 16314434 | 2005 | The Journal of experimental medicine |
| Itgax | 58 | 15944318 | 2005 | Journal of immunology (Baltimore, Md. : 1950) |
| Itgax | 58 | 15781587 | 2005 | The Journal of experimental medicine |
| Itgax | 58 | 15196283 | 2004 | Clinical and experimental allergy |
| Itgax | 58 | 15096186 | 2004 | Immunology |
| Itgax | 58 | 12702544 | 2003 | American journal of respiratory cell and molecular biology |
| Itgax | 58 | 12603602 | 2003 | Immunology |
| Itgax | 58 | 12393720 | 2002 | Blood |
| Itgax | 58 | 11869687 | 2002 | Immunity |
| Itgax | 58 | 10688435 | 1999 | Allergy |
| Itgax | 58 | 9450145 | 1997 | Allergy |
| Itgax | 58 | 7596088 | 1995 | The Kurume medical journal |
| Il1rn | 34 | 21622869 | 2011 | Journal of immunology (Baltimore, Md. : 1950) |
| Il1rn | 34 | 21252117 | 2011 | Carcinogenesis |
| Il1rn | 34 | 20523065 | 2010 | International archives of allergy and immunology |
| Il1rn | 34 | 19768973 | NA | Revista alergia Mexico (Tecamachalco, Puebla, Mexico : 1993) |
| Il1rn | 34 | 19149188 | 2008 | Chinese journal of biotechnology |
| Il1rn | 34 | 19087723 | 2008 | Zhonghua yi xue za zhi |
| Il1rn | 34 | 18959005 | 2008 | Journal of molecular cell biology |
| Il1rn | 34 | 18926055 | NA | Allergy and asthma proceedings |
| Il1rn | 34 | 18810365 | 2008 | Indian journal of pediatrics |
| Il1rn | 34 | 17116976 | 2006 | Journal of pharmacological sciences |
| Il1rn | 34 | 17107994 | 2007 | The European respiratory journal |
| Il1rn | 34 | 17021861 | 2006 | Immunogenetics |
| Il1rn | 34 | 16724092 | 2006 | Gene therapy |
| Il1rn | 34 | 16519819 | 2006 | BMC medical genetics |
| Il1rn | 34 | 16409203 | 2006 | Allergy |
| Il1rn | 34 | 15539764 | 2005 | Biology of the neonate |
| Il1rn | 34 | 15020290 | 2004 | American journal of respiratory and critical care medicine |
| Il1rn | 34 | 14730914 | 2003 | Yao xue xue bao = Acta pharmaceutica Sinica |
| Il1rn | 34 | 14519149 | 2003 | Clinical and experimental allergy |
| Il1rn | 34 | 12938145 | 2003 | Journal of clinical laboratory analysis |
| Il1rn | 34 | 12663678 | 2003 | International immunology |
| Il1rn | 34 | 12467523 | 2002 | Mediators of inflammation |
| Il1rn | 34 | 11360527 | 1998 | Chinese journal of tuberculosis and respiratory diseases |
| Il1rn | 34 | 11027520 | 2000 | Biochemical and biophysical research communications |
| Il1rn | 34 | 10843772 | 2000 | Cytokine |
| Il1rn | 34 | 10667111 | NA | ThÃ©rapie |
| Il1rn | 34 | 10487780 | 1999 | The Journal of clinical investigation |
| Il1rn | 34 | 9949321 | 1999 | The Journal of allergy and clinical immunology |
| Il1rn | 34 | 9927362 | 1999 | American journal of respiratory and critical care medicine |
| Il1rn | 34 | 9811535 | 1998 | Cytokine |
| Il1rn | 34 | 8887608 | 1996 | American journal of respiratory and critical care medicine |
| Il1rn | 34 | 8870701 | 1996 | Clinical and experimental immunology |
| Il1rn | 34 | 7631820 | 1995 | The American journal of physiology |
| Il1rn | 34 | 8038709 | 1994 | Receptor |
| Chi3l1 | 35 | 23190377 | 2012 | The Journal of asthma |
| Chi3l1 | 35 | 22857879 | 2012 | The Journal of allergy and clinical immunology |
| Chi3l1 | 35 | 22742450 | 2012 | The Biochemical journal |
| Chi3l1 | 35 | 22554524 | 2012 | Biochemical and biophysical research communications |
| Chi3l1 | 35 | 22550243 | 2012 | Proceedings of the American Thoracic Society |
| Chi3l1 | 35 | 22534532 | 2012 | The Journal of allergy and clinical immunology |
| Chi3l1 | 35 | 22480951 | 2012 | Cytokine |
| Chi3l1 | 35 | 22281830 | 2012 | American journal of respiratory and critical care medicine |
| Chi3l1 | 35 | 21968467 | 2012 | Respiration; international review of thoracic diseases |
| Chi3l1 | 35 | 21949714 | 2011 | PloS one |
| Chi3l1 | 35 | 21899483 | 2011 | The Journal of asthma |
| Chi3l1 | 35 | 21530869 | 2011 | Annals of allergy, asthma & immunology |
| Chi3l1 | 35 | 21159721 | 2011 | Multiple sclerosis (Houndmills, Basingstoke, England) |
| Chi3l1 | 35 | 21054166 | 2011 | Annual review of physiology |
| Chi3l1 | 35 | 20650887 | 2010 | The Journal of biological chemistry |
| Chi3l1 | 35 | 20538957 | 2010 | American journal of respiratory and critical care medicine |
| Chi3l1 | 35 | 20356987 | 2010 | The European respiratory journal |
| Chi3l1 | 35 | 20347285 | 2010 | Respiratory medicine |
| Chi3l1 | 35 | 20226308 | 2010 | The Journal of allergy and clinical immunology |
| Chi3l1 | 35 | 20224674 | 2010 | Allergy, asthma & immunology research |
| Chi3l1 | 35 | 19908331 | 2009 | World journal of gastroenterology : WJG |
| Chi3l1 | 35 | 19644363 | 2009 | Current opinion in allergy and clinical immunology |
| Chi3l1 | 35 | 19568425 | 2009 | PloS one |
| Chi3l1 | 35 | 19532094 | 2009 | Current opinion in allergy and clinical immunology |
| Chi3l1 | 35 | 19421404 | 2009 | PloS one |
| Chi3l1 | 35 | 19414556 | 2009 | The Journal of experimental medicine |
| Chi3l1 | 35 | 18769193 | 2008 | Current opinion in allergy and clinical immunology |
| Chi3l1 | 35 | 18498542 | 2008 | Clinical and experimental allergy |
| Chi3l1 | 35 | 18403760 | 2008 | The New England journal of medicine |
| Chi3l1 | 35 | 18403759 | 2008 | The New England journal of medicine |
| Chi3l1 | 35 | 18334164 | 2008 | MÃ©decine sciences : M/S |
| Chi3l1 | 35 | 18322291 | 2008 | The New England journal of medicine |
| Chi3l1 | 35 | 18003958 | 2007 | The New England journal of medicine |
| Chi3l1 | 35 | 17709146 | 2007 | Veterinary immunology and immunopathology |
| Chi3l1 | 35 | 17392594 | 2007 | The Keio journal of medicine |
| Cd68 | 55 | 21943944 | 2012 | The Journal of allergy and clinical immunology |
| Cd68 | 55 | 21681974 | 2011 | Diagnostic cytopathology |
| Cd68 | 55 | 21512269 | 2011 | Experimental animals / Japanese Association for Laboratory Animal Science |
| Cd68 | 55 | 20644177 | 2010 | Journal of immunology (Baltimore, Md. : 1950) |
| Cd68 | 55 | 19961259 | 2009 | Archives of pathology & laboratory medicine |
| Cd68 | 55 | 19490801 | NA | American journal of rhinology & allergy |
| Cd68 | 55 | 19191129 | 2009 | The Journal of asthma |
| Cd68 | 55 | 19132974 | 2009 | Allergy |
| Cd68 | 55 | 18681853 | 2008 | Clinical and experimental allergy |
| Cd68 | 55 | 18498542 | 2008 | Clinical and experimental allergy |
| Cd68 | 55 | 18268925 | 2007 | International journal of chronic obstructive pulmonary disease |
| Cd68 | 55 | 18250182 | 2008 | Thorax |
| Cd68 | 55 | 17441790 | 2007 | Allergy |
| Cd68 | 55 | 17272787 | 2007 | American journal of respiratory and critical care medicine |
| Cd68 | 55 | 17075272 | 2006 | Allergology international |
| Cd68 | 55 | 17035437 | 2006 | Chest |
| Cd68 | 55 | 16959617 | 2006 | Journal of the Formosan Medical Association = Taiwan yi zhi |
| Cd68 | 55 | 16899487 | 2006 | The European respiratory journal |
| Cd68 | 55 | 15805998 | 2005 | The Journal of allergy and clinical immunology |
| Cd68 | 55 | 15784110 | 2005 | Clinical and experimental allergy |
| Cd68 | 55 | 15510586 | NA | Allergy and asthma proceedings |
| Cd68 | 55 | 14564360 | 2003 | The Journal of allergy and clinical immunology |
| Cd68 | 55 | 12212952 | 2002 | The European respiratory journal |
| Cd68 | 55 | 12149529 | 2002 | Thorax |
| Cd68 | 55 | 12070058 | 2002 | American journal of respiratory and critical care medicine |
| Cd68 | 55 | 12028114 | 2002 | Allergy |
| Cd68 | 55 | 11972605 | 2002 | Clinical and experimental allergy |
| Cd68 | 55 | 11502664 | 2001 | Chest |
| Cd68 | 55 | 11199094 | 2001 | Novartis Foundation symposium |
| Cd68 | 55 | 11182013 | 2001 | Thorax |
| Cd68 | 55 | 11069834 | 2000 | American journal of respiratory and critical care medicine |
| Cd68 | 55 | 11031340 | 2000 | The Journal of allergy and clinical immunology |
| Cd68 | 55 | 10984367 | 2000 | The Journal of allergy and clinical immunology |
| Cd68 | 55 | 10843939 | 2000 | Chest |
| Cd68 | 55 | 10573219 | 1999 | The European respiratory journal |
| Cd68 | 55 | 10570327 | 1999 | Journal of immunology (Baltimore, Md. : 1950) |
| Cd68 | 55 | 10556105 | 1999 | American journal of respiratory and critical care medicine |
| Cd68 | 55 | 10394103 | 1999 | International archives of allergy and immunology |
| Cd68 | 55 | 10362043 | 1999 | The European respiratory journal |
| Cd68 | 55 | 10193369 | 1998 | Thorax |
| Cd68 | 55 | 9257786 | 1997 | The Journal of allergy and clinical immunology |
| Cd68 | 55 | 9155834 | 1997 | The Journal of allergy and clinical immunology |
| Cd68 | 55 | 9150325 | 1997 | The European respiratory journal |
| Cd68 | 55 | 9117016 | 1997 | American journal of respiratory and critical care medicine |
| Cd68 | 55 | 8970374 | 1996 | American journal of respiratory and critical care medicine |
| Cd68 | 55 | 8887608 | 1996 | American journal of respiratory and critical care medicine |
| Cd68 | 55 | 8680684 | 1996 | American journal of respiratory and critical care medicine |
| Cd68 | 55 | 8630259 | 1996 | American journal of respiratory cell and molecular biology |
| Cd68 | 55 | 7668997 | 1995 | ArerugÄ« = [Allergy] |
| Cd68 | 55 | 7742012 | 1995 | American journal of respiratory cell and molecular biology |
| Cd68 | 55 | 7613132 | NA | International archives of allergy and immunology |
| Cd68 | 55 | 7874318 | NA | Journal of investigational allergology & clinical immunology |
| Cd68 | 55 | 8417755 | 1993 | American journal of respiratory cell and molecular biology |
| Cd68 | 55 | 1489147 | 1992 | The American review of respiratory disease |
| Cd68 | 55 | 1532807 | 1992 | The Journal of allergy and clinical immunology |
| Gatm | 0 | 0 |  |  |
| Olfm1 | 0 | 0 |  |  |
| Sirpa | 0 | 0 |  |  |
| Ptgs1 | 65 | 22829846 | 2012 | Journal of allergy |
| Ptgs1 | 65 | 22627848 | 2012 | Allergology international |
| Ptgs1 | 65 | 22484053 | 2012 | Journal of ethnopharmacology |
| Ptgs1 | 65 | 22324934 | 2012 | Expert opinion on therapeutic targets |
| Ptgs1 | 65 | 22132000 | 2012 | Journal of allergy |
| Ptgs1 | 65 | 21331560 | 2011 | Journal of pharmacokinetics and pharmacodynamics |
| Ptgs1 | 65 | 21039786 | 2010 | The Journal of dermatology |
| Ptgs1 | 65 | 20955151 | 2011 | Current drug targets |
| Ptgs1 | 65 | 20656922 | 2010 | Journal of immunology (Baltimore, Md. : 1950) |
| Ptgs1 | 65 | 20633623 | 2011 | Journal of ethnopharmacology |
| Ptgs1 | 65 | 20631417 | NA | Pharmacological reports : PR |
| Ptgs1 | 65 | 20519889 | 2010 | Chemical immunology and allergy |
| Ptgs1 | 65 | 19879442 | 2009 | Immunology and allergy clinics of North America |
| Ptgs1 | 65 | 19575683 | 2009 | Annual review of physiology |
| Ptgs1 | 65 | 19251796 | 2009 | The European respiratory journal Physiology |
| Ptgs1 | 65 | 19132974 | 2009 | Allergy |
| Ptgs1 | 65 | 18996575 | 2009 | The Journal of allergy and clinical immunology |
| Ptgs1 | 65 | 18753249 | 2008 | FASEB journal |
| Ptgs1 | 65 | 17609236 | 2007 | The Annals of pharmacotherapy |
| Ptgs1 | 65 | 17508966 | 2007 | Allergy |
| Ptgs1 | 65 | 17454003 | 2007 | DNA sequence |
| Ptgs1 | 65 | 17292954 | 2007 | The Journal of allergy and clinical immunology |
| Ptgs1 | 65 | 16579869 | 2006 | Current allergy and asthma reports |
| Ptgs1 | 65 | 16457808 | 2006 | European journal of pharmacology |
| Ptgs1 | 65 | 16433444 | 2005 | Bulletin de l'AcadÃ©mie nationale de mÃ©decine |
| Ptgs1 | 65 | 16055872 | 2005 | The European respiratory journal |
| Ptgs1 | 65 | 15613671 | 2004 | JAMA : the journal of the American Medical Association |
| Ptgs1 | 65 | 15521369 | 2004 | Annals of allergy, asthma & immunology munology |
| Ptgs1 | 65 | 15480320 | 2004 | The Journal of allergy and clinical immunology |
| Ptgs1 | 65 | 15375155 | 2004 | The Journal of biological chemistry |
| Ptgs1 | 65 | 15242723 | 2004 | Immunology and allergy clinics of North America |
| Ptgs1 | 65 | 15151923 | 2004 | American journal of respiratory and critical care medicine |
| Ptgs1 | 65 | 15100686 | 2004 | The Journal of allergy and clinical immunology |
| Ptgs1 | 65 | 14769263 | 2004 | Current allergy and asthma reports |
| Ptgs1 | 65 | 14680616 | 2004 | Current allergy and asthma reports |
| Ptgs1 | 65 | 14600429 | 2003 | International archives of allergy and immunology |
| Ptgs1 | 65 | 14561202 | 2002 | Current drug targets. Inflammation and allergy |
| Ptgs1 | 65 | 14552699 | NA | International journal of immunopathology and pharmacology |
| Ptgs1 | 65 | 12895598 | NA | Prostaglandins, leukotrienes, and essential fatty acids |
| Ptgs1 | 65 | 12796206 | 2003 | Chest |
| Ptgs1 | 65 | 12743569 | 2003 | The Journal of allergy and clinical immunology |
| Ptgs1 | 65 | 12668895 | 2003 | Clinical reviews in allergy & immunology |
| Ptgs1 | 65 | 12576199 | 2003 | Journal of ethnopharmacology |
| Ptgs1 | 65 | 12529163 | 2003 | Paediatric drugs |
| Ptgs1 | 65 | 12487218 | 2002 | Annals of allergy, asthma & immunology |
| Ptgs1 | 65 | 12429575 | 2002 | British journal of pharmacology |
| Ptgs1 | 65 | 11952135 | 2002 | Viral immunology |
| Ptgs1 | 65 | 11943670 | 2002 | American journal of physiology. Lung cellular and molecular physiology |
| Ptgs1 | 65 | 11940059 | 2002 | Clinical and experimental allergy |
| Ptgs1 | 65 | 11860351 | 2002 | Current medicinal chemistry |
| Ptgs1 | 65 | 11694451 | 2001 | American journal of respiratory cell and molecular biology |
| Ptgs1 | 65 | 11447381 | 2001 | The Journal of allergy and clinical immunology |
| Ptgs1 | 65 | 11394934 | 2001 | Pharmacological research |
| Ptgs1 | 65 | 11273789 | 2001 | Pulmonary pharmacology & therapeutics |
| Ptgs1 | 65 | 11251623 | 2001 | Clinical and experimental allergy |
| Ptgs1 | 65 | 11251618 | 2001 | Clinical and experimental allergy |
| Ptgs1 | 65 | 11237998 | 2001 | American journal of physiology. Lung cellular and molecular physiology |
| Ptgs1 | 65 | 11152649 | 2001 | American journal of respiratory cell and molecular biology |
| Ptgs1 | 65 | 10992560 | 2000 | Thorax |
| Ptgs1 | 65 | 10400832 | 1999 | The Journal of allergy and clinical immunology |
| Ptgs1 | 65 | 10390414 | 1999 | American journal of respiratory and critical care medicine |
| Ptgs1 | 65 | 9846651 | 1998 | British journal of pharmacology |
| Ptgs1 | 65 | 9761007 | 1998 | Clinical and experimental allergy |
| Ptgs1 | 65 | 9416556 | NA | Journal of investigational allergology & clinical immunology |
| Ptgs1 | 65 | 9032211 | 1997 | American journal of respiratory and critical care medicine |
| Ccl9 | 4 | 20622891 | 2010 | Cellular & molecular immunology |
| Ccl9 | 4 | 16339523 | 2005 | Journal of immunology (Baltimore, Md. : 1950) |
| Ccl9 | 4 | 15585884 | 2004 | Journal of immunology (Baltimore, Md. : 1950) |
| Ccl9 | 4 | 15203102 | 2004 | The international journal of biochemistry & cell biology |
| Ccl6 | 8 | 20622891 | 2010 | Cellular & molecular immunology |
| Ccl6 | 8 | 18156208 | 2008 | The American journal of pathology |
| Ccl6 | 8 | 17168792 | 2006 | Inflammation & allergy drug targets |
| Ccl6 | 8 | 16645178 | 2006 | American journal of respiratory cell and molecular biology |
| Ccl6 | 8 | 16607380 | 2006 | Laboratory investigation; a journal of technical methods and pathology |
| Ccl6 | 8 | 16251377 | 2005 | Occupational medicine (Oxford, England) |
| Ccl6 | 8 | 15585884 | 2004 | Journal of immunology (Baltimore, Md. : 1950) |
| Ccl6 | 8 | 15374841 | 2005 | American journal of respiratory and critical care medicine |
| 1100001G20Rik | 0 | 0 |  |  |
| Bpifb1 | 0 | 0 |  |  |
| Procr | 1 | 14604971 | 2004 | Blood |
| Lbp | 10 | 23063165 | 2012 | The Journal of allergy and clinical immunology |
| Lbp | 10 | 20226507 | 2010 | The Journal of allergy and clinical immunology |
| Lbp | 10 | 16740168 | 2006 | BMC pulmonary medicine |
| Lbp | 10 | 16539740 | 2006 | BMC musculoskeletal disorders |
| Lbp | 10 | 15356561 | 2004 | The Journal of allergy and clinical immunology |
| Lbp | 10 | 12397021 | 2002 | American journal of respiratory cell and molecular biology |
| Lbp | 10 | 11181108 | 2001 | Toxicology and applied pharmacology |
| Lbp | 10 | 11160257 | 2001 | Journal of immunology (Baltimore, Md. : 1950) |
| Lbp | 10 | 8967507 | 1996 | The American journal of physiology |
| Lbp | 10 | 8838085 | 1996 | Nihon rinsho. Japanese journal of clinical medicine |
| Rab20 | 0 | 0 |  |  |
| F10 | 4 | 22970026 | 2012 | Experimental and therapeutic medicine |
| F10 | 4 | 21892786 | 2012 | Mycopathologia |
| F10 | 4 | 18922934 | 2008 | Cancer research |
| F10 | 4 | 16086832 | 2005 | Respiratory research |
| Naip2 | 0 | 0 |  |  |
| Cd14 | 179 | 23194293 | 2012 | Pediatric allergy and immunology |
| Cd14 | 179 | 23101184 | 2012 | Journal of investigational allergology & clinical immunology |
| Cd14 | 179 | 22697010 | 2012 | Journal of investigational allergology & clinical immunology |
| Cd14 | 179 | 22564189 | 2012 | Allergy |
| Cd14 | 179 | 22377711 | 2012 | Journal of human genetics |
| Cd14 | 179 | 22376040 | 2012 | The Journal of asthma |
| Cd14 | 179 | 22356142 | 2012 | Clinical and experimental allergy |
| Cd14 | 179 | 22328887 | 2011 | Archives of medical science : AMS |
| Cd14 | 179 | 22299310 | 2011 | Asian Pacific journal of allergy and immunology |
| Cd14 | 179 | 22032786 | 2011 | International journal of immunopathology and pharmacology |
| Cd14 | 179 | 22015088 | 2011 | Respiratory medicine |
| Cd14 | 179 | 21905503 | 2011 | Journal of investigational allergology & clinical immunology |
| Cd14 | 179 | 21842127 | NA | Molecular medicine reports |
| Cd14 | 179 | 21749458 | 2011 | Pediatric allergy and immunology |
| Cd14 | 179 | 21745379 | 2011 | BMC medical genetics |
| Cd14 | 179 | 21646795 | 2011 | International archives of allergy and immunology |
| Cd14 | 179 | 21489615 | 2011 | The Journal of allergy and clinical immunology |
| Cd14 | 179 | 21389010 | 2011 | Occupational and environmental medicine |
| Cd14 | 179 | 21325943 | 2011 | Current opinion in allergy and clinical immunology |
| Cd14 | 179 | 21324477 | 2011 | The Journal of pediatrics |
| Cd14 | 179 | 21274737 | 2011 | Inflammation research |
| Cd14 | 179 | 21079949 | 2011 | Immunogenetics |
| Cd14 | 179 | 21039977 | 2010 | Clinical and experimental allergy |
| Cd14 | 179 | 21039600 | 2011 | Allergy |
| Cd14 | 179 | 20726961 | 2011 | Allergy |
| Cd14 | 179 | 20701615 | 2010 | Clinical and experimental allergy |
| Cd14 | 179 | 20618347 | 2010 | Clinical and experimental allergy |
| Cd14 | 179 | 20608916 | 2011 | Allergy |
| Cd14 | 179 | 20579716 | 2010 | The Journal of allergy and clinical immunology |
| Cd14 | 179 | 20574656 | 2010 | Human genetics |
| Cd14 | 179 | 20536280 | 2010 | The Journal of asthma |
| Cd14 | 179 | 20398919 | 2010 | The Journal of allergy and clinical immunology |
| Cd14 | 179 | 20394509 | 2010 | The Journal of asthma |
| Cd14 | 179 | 20384875 | 2010 | Scandinavian journal of immunology |
| Cd14 | 179 | 20302606 | 2010 | Respiratory research |
| Cd14 | 179 | 20179765 | 2010 | PloS one |
| Cd14 | 179 | 20126925 | 2009 | Jornal brasileiro de pneumologia |
| Cd14 | 179 | 20085599 | 2009 | Clinical and experimental allergy |
| Cd14 | 179 | 20080799 | 2010 | Proceedings of the National Academy of Sciences of the United States of America |
| Cd14 | 179 | 20051845 | 2010 | Current opinion in allergy and clinical immunology |
| Cd14 | 179 | 19968655 | 2010 | Clinical and experimental allergy |
| Cd14 | 179 | 19883332 | 2009 | Expert review of anti-infective therapy |
| Cd14 | 179 | 19825525 | 2009 | European cytokine network |
| Cd14 | 179 | 19796192 | 2010 | Allergy |
| Cd14 | 179 | 19785013 | 2009 | Stem cells (Dayton, Ohio) |
| Cd14 | 179 | 19462345 | 2009 | Pneumonologia i alergologia polska |
| Cd14 | 179 | 19372244 | 2010 | American journal of respiratory cell and molecular biology |
| Cd14 | 179 | 19361972 | 2009 | Respiratory medicine |
| Cd14 | 179 | 19254290 | 2009 | Allergy |
| Cd14 | 179 | 19222419 | 2009 | Allergy |
| Cd14 | 179 | 19191129 | 2009 | The Journal of asthma |
| Cd14 | 179 | 19148143 | 2009 | Genes and immunity |
| Cd14 | 179 | 19119705 | 2008 | Annals of allergy, asthma & immunology |
| Cd14 | 179 | 19109137 | 2009 | Journal of immunology (Baltimore, Md. : 1950) |
| Cd14 | 179 | 19096003 | 2009 | American journal of respiratory and critical care medicine |
| Cd14 | 179 | 18952503 | 2009 | Clinical immunology (Orlando, Fla.) |
| Cd14 | 179 | 18931892 | 2009 | Journal of clinical immunology |
| Cd14 | 179 | 18774388 | 2008 | The Journal of allergy and clinical immunology |
| Cd14 | 179 | 18714537 | 2008 | Journal of investigational allergology & clinical immunology |
| Cd14 | 179 | 18446588 | 2008 | The Journal of asthma : official journal of the Association for the Care of Asthma |
| Cd14 | 179 | 18426139 | 2008 | Annals of allergy, asthma & immunology |
| Cd14 | 179 | 18425216 | NA | Jornal de pediatria |
| Cd14 | 179 | 18417506 | 2008 | The European respiratory journal |
| Cd14 | 179 | 18312481 | 2008 | Tissue antigens |
| Cd14 | 179 | 17989521 | 2007 | Current opinion in allergy and clinical immunology |
| Cd14 | 179 | 17954484 | 2007 | Archives of disease in childhood |
| Cd14 | 179 | 17951166 | 2007 | Folia histochemica et cytobiologica |
| Cd14 | 179 | 17919709 | 2007 | The Journal of allergy and clinical immunology |
| Cd14 | 179 | 17910328 | 2007 | Annals of allergy, asthma & immunology |
| Cd14 | 179 | 17877764 | 2007 | Clinical and experimental allergy |
| Cd14 | 179 | 17823973 | 2007 | Human mutation |
| Cd14 | 179 | 17607003 | 2007 | Proceedings of the American Thoracic Society |
| Cd14 | 179 | 17581203 | 2007 | Clinical and experimental allergy |
| Cd14 | 179 | 17574828 | 2007 | Respiratory medicine |
| Cd14 | 179 | 17456337 | 2007 | Zhonghua er ke za zhi. Chinese journal of pediatrics |
| Cd14 | 179 | 17349684 | 2007 | The Journal of allergy and clinical immunology |
| Cd14 | 179 | 17270707 | 2007 | Immunobiology |
| Cd14 | 179 | 17218815 | 2007 | Current opinion in allergy and clinical immunology |
| Cd14 | 179 | 17202288 | 2007 | Proceedings of the American Thoracic Society |
| Cd14 | 179 | 17201240 | 2006 | Annals of allergy, asthma & immunology |
| Cd14 | 179 | 17196641 | 2007 | The Journal of allergy and clinical immunology |
| Cd14 | 179 | 17175987 | 2006 | Pneumonologia i alergologia polska |
| Cd14 | 179 | 17083354 | 2006 | Clinical and experimental allergy |
| Cd14 | 179 | 17075287 | 2006 | Allergology international |
| Cd14 | 179 | 17042137 | 2006 | Annals of allergy, asthma & immunology |
| Cd14 | 179 | 17003960 | 2006 | Journal of human genetics |
| Cd14 | 179 | 16959617 | 2006 | Journal of the Formosan Medical Association |
| Cd14 | 179 | 16954783 | 2006 | Current opinion in allergy and clinical immunology |
| Cd14 | 179 | 16844729 | 2007 | Thorax |
| Cd14 | 179 | 16815140 | 2006 | The Journal of allergy and clinical immunology |
| Cd14 | 179 | 16771785 | 2006 | Pediatric allergy and immunology |
| Cd14 | 179 | 16630939 | 2006 | The Journal of allergy and clinical immunology |
| Cd14 | 179 | 16566859 | 2006 | Current allergy and asthma reports |
| Cd14 | 179 | 16543402 | 2006 | Journal of leukocyte biology |
| Cd14 | 179 | 16505608 | 2006 | Current opinion in allergy and clinical immunology |
| Cd14 | 179 | 16446545 | 2006 | International archives of allergy and immunology |
| Cd14 | 179 | 16446543 | 2006 | International archives of allergy and immunology |
| Cd14 | 179 | 16387800 | 2006 | American journal of respiratory and critical care medicine |
| Cd14 | 179 | 16310521 | 2006 | The Medical clinics of North America |
| Cd14 | 179 | 16266379 | 2005 | Allergy |
| Cd14 | 179 | 16266378 | 2005 | Allergy |
| Cd14 | 179 | 16257634 | 2005 | Immunology and allergy clinics of North America |
| Cd14 | 179 | 16214776 | 2005 | Acta paediatrica (Oslo, Norway : 1992). Supplement |
| Cd14 | 179 | 16202577 | 2006 | Respiratory medicine |
| Cd14 | 179 | 16120082 | 2005 | Clinical and experimental allergy |
| Cd14 | 179 | 15992841 | 2005 | Toxicology and applied pharmacology |
| Cd14 | 179 | 15940135 | 2005 | The Journal of allergy and clinical immunology |
| Cd14 | 179 | 15897161 | 2005 | The Journal of dermatological treatment |
| Cd14 | 179 | 15879416 | 2005 | American journal of respiratory and critical care medicine |
| Cd14 | 179 | 15853738 | 2005 | Current drug targets. Inflammation and allergy |
| Cd14 | 179 | 15753897 | 2005 | The Journal of allergy and clinical immunology |
| Cd14 | 179 | 15741437 | 2005 | Thorax |
| Cd14 | 179 | 15683456 | 2005 | Scandinavian journal of immunology |
| Cd14 | 179 | 15660518 | 2005 | Annual review of medicine |
| Cd14 | 179 | 15649267 | 2005 | Clinical and experimental allergy |
| Cd14 | 179 | 15602630 | 2005 | Journal of human genetics |
| Cd14 | 179 | 15378299 | 2004 | Immunogenetics |
| Cd14 | 179 | 15356557 | 2004 | The Journal of allergy and clinical immunology |
| Cd14 | 179 | 15281474 | 2004 | Annals of allergy, asthma & immunology |
| Cd14 | 179 | 15241347 | 2004 | The Journal of allergy and clinical immunology |
| Cd14 | 179 | 15191023 | 2004 | Annals of allergy, asthma & immunology |
| Cd14 | 179 | 15136573 | 2004 | The Journal of biological chemistry |
| Cd14 | 179 | 15007332 | 2004 | The Journal of allergy and clinical immunology |
| Cd14 | 179 | 14749527 | 2003 | Experimental & molecular medicine |
| Cd14 | 179 | 14641542 | 2003 | European journal of immunogenetics |
| Cd14 | 179 | 14517492 | 2003 | Blood coagulation & fibrinolysis |
| Cd14 | 179 | 14510720 | 2003 | Allergy |
| Cd14 | 179 | 14501431 | 2003 | Current opinion in allergy and clinical immunology |
| Cd14 | 179 | 12911501 | 2003 | Pediatric allergy and immunology |
| Cd14 | 179 | 12897754 | 2003 | The Journal of allergy and clinical immunology |
| Cd14 | 179 | 12760962 | 2003 | American journal of respiratory cell and molecular biology |
| Cd14 | 179 | 12743572 | 2003 | The Journal of allergy and clinical immunology |
| Cd14 | 179 | 12722945 | 2003 | Immunological investigations |
| Cd14 | 179 | 12680871 | 2003 | Clinical and experimental allergy |
| Cd14 | 179 | 12580907 | 2003 | Clinical and experimental allergy |
| Cd14 | 179 | 12449174 | 2002 | The European respiratory journal |
| Cd14 | 179 | 12397021 | 2002 | American journal of respiratory cell and molecular biology |
| Cd14 | 179 | 12011764 | 2002 | Medical science monitor |
| Cd14 | 179 | 11972599 | 2002 | Clinical and experimental allergy |
| Cd14 | 179 | 11964695 | 2001 | Current opinion in allergy and clinical immunology |
| Cd14 | 179 | 11936536 | 2002 | The European respiratory journal |
| Cd14 | 179 | 11890712 | 2002 | Clinical immunology (Orlando, Fla.) |
| Cd14 | 179 | 11753119 | 2002 | Current opinion in pulmonary medicine |
| Cd14 | 179 | 11732288 | 2001 | Pneumonologia i alergologia polska |
| Cd14 | 179 | 11590384 | 2001 | The Journal of allergy and clinical immunology |
| Cd14 | 179 | 11574751 | 2001 | International archives of allergy and immunology |
| Cd14 | 179 | 11521081 | 2001 | Journal of endotoxin research |
| Cd14 | 179 | 11398078 | 2001 | The Journal of allergy and clinical immunology |
| Cd14 | 179 | 11359627 | 2000 | British medical bulletin |
| Cd14 | 179 | 11306916 | NA | International archives of allergy and immunology |
| Cd14 | 179 | 11282774 | 2001 | American journal of respiratory and critical care medicine |
| Cd14 | 179 | 11278629 | 2001 | The Journal of biological chemistry |
| Cd14 | 179 | 11159017 | 2001 | American journal of physiology. Lung cellular and molecular physiology |
| Cd14 | 179 | 11104731 | 2000 | American journal of respiratory cell and molecular biology |
| Cd14 | 179 | 11090937 | 2000 | Toxicology |
| Cd14 | 179 | 11022011 | 2000 | American journal of human genetics |
| Cd14 | 179 | 10919504 | 2000 | Allergy |
| Cd14 | 179 | 10907586 | 2000 | Clinics in chest medicine |
| Cd14 | 179 | 10809960 | 2000 | Immunology |
| Cd14 | 179 | 10804928 | 2000 | The Israel Medical Association journal : IMAJ |
| Cd14 | 179 | 10719296 | 2000 | The Journal of allergy and clinical immunology |
| Cd14 | 179 | 10631542 | 1999 | Current opinion in immunology |
| Cd14 | 179 | 10594539 | 1999 | Clinical and experimental allergy |
| Cd14 | 179 | 10587479 | 1999 | Pulmonary pharmacology & therapeutics |
| Cd14 | 179 | 10432289 | 1999 | The Journal of experimental medicine |
| Cd14 | 179 | 10069865 | 1999 | The Journal of allergy and clinical immunology |
| Cd14 | 179 | 9890612 | 1999 | Pediatric research |
| Cd14 | 179 | 9561931 | 1998 | Inflammation |
| Cd14 | 179 | 9551731 | 1998 | The European respiratory journal |
| Cd14 | 179 | 9450145 | 1997 | Allergy |
| Cd14 | 179 | 9212832 | 1997 | Journal of immunological methods |
| Cd14 | 179 | 9117017 | 1997 | American journal of respiratory and critical care medicine |
| Cd14 | 179 | 8967507 | 1996 | The American journal of physiology |
| Cd14 | 179 | 18475731 | 1996 | Mediators of inflammation |
| Cd14 | 179 | 7767539 | 1995 | American journal of respiratory and critical care medicine |
| Cd14 | 179 | 7812576 | 1995 | American journal of respiratory and critical care medicine |
| Cd14 | 179 | 8207248 | 1994 | Journal of immunology (Baltimore, Md. : 1950) |
| Cd14 | 179 | 8173641 | 1994 | Pediatric allergy and immunology |
| Cd14 | 179 | 8386056 | 1993 | Monaldi archives for chest disease |
| Cd200r1 | 0 | 0 |  |  |
| Mtm1 | 0 | 0 |  |  |
| Tlr7 | 36 | 23078048 | 2012 | Inflammation & allergy drug targets |
| Tlr7 | 36 | 22882449 | 2012 | Allergy |
| Tlr7 | 36 | 22857391 | 2012 | BMC medical genetics |
| Tlr7 | 36 | 22727330 | 2012 | Immunobiology |
| Tlr7 | 36 | 22657407 | 2012 | The Journal of allergy and clinical immunology |
| Tlr7 | 36 | 22491246 | 2012 | Journal of immunology (Baltimore, Md. : 1950) |
| Tlr7 | 36 | 22355409 | 2012 | PloS one |
| Tlr7 | 36 | 22125636 | 2011 | PloS one |
| Tlr7 | 36 | 22086297 | 2012 | Current allergy and asthma reports |
| Tlr7 | 36 | 22035076 | 2012 | British journal of pharmacology |
| Tlr7 | 36 | 21917654 | 2012 | Thorax |
| Tlr7 | 36 | 21748646 | 2011 | Methods in molecular biology (Clifton, N.J.) |
| Tlr7 | 36 | 21646801 | 2011 | International archives of allergy and immunology |
| Tlr7 | 36 | 21480211 | 2011 | European journal of immunology |
| Tlr7 | 36 | 21460120 | 2011 | American journal of physiology. Lung cellular and molecular physiology |
| Tlr7 | 36 | 21389257 | 2011 | Journal of immunology (Baltimore, Md. : 1950) |
| Tlr7 | 36 | 21375463 | 2011 | Expert opinion on therapeutic targets |
| Tlr7 | 36 | 21364926 | 2011 | PloS one |
| Tlr7 | 36 | 21335488 | 2011 | Journal of immunology (Baltimore, Md. : 1950) |
| Tlr7 | 36 | 21167577 | 2011 | The Journal of allergy and clinical immunology |
| Tlr7 | 36 | 21157038 | 2011 | The Journal of clinical investigation |
| Tlr7 | 36 | 21131420 | 2011 | Journal of immunology (Baltimore, Md. : 1950) |
| Tlr7 | 36 | 20412137 | 2010 | Clinical and experimental allergy |
| Tlr7 | 36 | 20410486 | 2010 | Journal of immunology (Baltimore, Md. : 1950) |
| Tlr7 | 36 | 20377514 | 2010 | Current medicinal chemistry |
| Tlr7 | 36 | 20224068 | 2010 | American journal of respiratory and critical care medicine |
| Tlr7 | 36 | 19735273 | 2009 | Clinical and experimental allergy |
| Tlr7 | 36 | 19643938 | 2010 | The European respiratory journal |
| Tlr7 | 36 | 19025588 | 2008 | Journal of neuroinflammation |
| Tlr7 | 36 | 18682521 | 2008 | Thorax |
| Tlr7 | 36 | 18220957 | 2007 | Inflammation & allergy drug targets |
| Tlr7 | 36 | 18031246 | 2007 | Biochemical Society transactions |
| Tlr7 | 36 | 18020622 | 2007 | BioDrugs : clinical immunotherapeutics, biopharmaceuticals and gene therapy |
| Tlr7 | 36 | 17548618 | 2007 | Journal of immunology (Baltimore, Md. : 1950) |
| Tlr7 | 36 | 17400732 | 2007 | American journal of respiratory and critical care medicine |
| Tlr7 | 36 | 16361354 | 2006 | American journal of physiology. Lung cellular and molecular physiology |
| Cybb | 4 | 22982469 | 2012 | Life sciences |
| Cybb | 4 | 17293377 | 2007 | American journal of physiology. Lung cellular and molecular physiology |
| Cybb | 4 | 16608528 | 2006 | Journal of negative results in biomedicine |
| Cybb | 4 | 14588148 | 2003 | Antioxidants & redox signaling |
| Atp6ap2 | 0 | 0 |  |  |
| Id2 | 0 | 0 |  |  |
| Slc26a4 | 9 | 22116372 | 2011 | Cellular physiology and biochemistry |
| Slc26a4 | 9 | 22116359 | 2011 | Cellular physiology and biochemistry |
| Slc26a4 | 9 | 22116352 | 2011 | Cellular physiology and biochemistry |
| Slc26a4 | 9 | 21814192 | 2011 | Clinical pharmacology and therapeutics |
| Slc26a4 | 9 | 21045265 | 2010 | Disease markers |
| Slc26a4 | 9 | 19289392 | 2009 | Journal of medical genetics |
| Slc26a4 | 9 | 19028979 | 2009 | American journal of physiology. Lung cellular and molecular physiology |
| Slc26a4 | 9 | 18641360 | 2008 | Journal of immunology (Baltimore, Md. : 1950) |
| Slc26a4 | 9 | 18424749 | 2008 | Journal of immunology (Baltimore, Md. : 1950) |
| Ms4a7 | 0 | 0 |  |  |
| Ms4a6d | 0 | 0 |  |  |
| Rab32 | 0 | 0 |  |  |
| Il33 | 45 | 23169007 | 2012 | European journal of immunology |
| Il33 | 45 | 22694930 | 2012 | The Journal of allergy and clinical immunology |
| Il33 | 45 | 22574108 | 2012 | PloS one |
| Il33 | 45 | 22562552 | 2012 | Applied biochemistry and biotechnology |
| Il33 | 45 | 22540331 | 2012 | Allergy |
| Il33 | 45 | 22349136 | 2012 | Inflammation research |
| Il33 | 45 | 22329990 | 2012 | The Journal of experimental medicine |
| Il33 | 45 | 22307629 | 2012 | Proceedings of the National Academy of Sciences of the United States of America |
| Il33 | 45 | 22233535 | 2012 | Clinical and experimental allergy |
| Il33 | 45 | 22215666 | 2012 | The Journal of biological chemistry |
| Il33 | 45 | 22112999 | 2012 | Current opinion in pulmonary medicine |
| Il33 | 45 | 21804549 | 2011 | Nature genetics |
| Il33 | 45 | 21802127 | 2011 | The Journal of allergy and clinical immunology |
| Il33 | 45 | 21712394 | 2011 | Journal of leukocyte biology |
| Il33 | 45 | 21682745 | 2011 | Immunological reviews |
| Il33 | 45 | 21682736 | 2011 | Immunological reviews |
| Il33 | 45 | 21629437 | 2010 | Current genomics |
| Il33 | 45 | 21519352 | 2011 | Nature reviews. Rheumatology |
| Il33 | 45 | 21301328 | 2011 | Current opinion in allergy and clinical immunology |
| Il33 | 45 | 21276132 | 2011 | Respirology (Carlton, Vic.) |
| Il33 | 45 | 21158975 | 2011 | Journal of internal medicine |
| Il33 | 45 | 21150435 | 2011 | Current opinion in allergy and clinical immunology |
| Il33 | 45 | 21071194 | 2010 | Current opinion in immunology |
| Il33 | 45 | 20931364 | 2011 | Current allergy and asthma reports |
| Il33 | 45 | 20926795 | 2010 | Journal of immunology (Baltimore, Md. : 1950) |
| Il33 | 45 | 20860503 | 2010 | The New England journal of medicine |
| Il33 | 45 | 20816195 | 2010 | The Journal of allergy and clinical immunology |
| Il33 | 45 | 20625511 | 2010 | PloS one |
| Il33 | 45 | 20608085 | 2010 | the journal of the Japanese Respiratory Society |
| Il33 | 45 | 20200520 | 2010 | Nature |
| Il33 | 45 | 20200518 | 2010 | Nature |
| Il33 | 45 | 20153038 | 2010 | The Journal of allergy and clinical immunology |
| Il33 | 45 | 20081870 | 2010 | Nature reviews. Immunology |
| Il33 | 45 | 20014018 | 2010 | Inflammatory bowel diseases |
| Il33 | 45 | 19906013 | 2010 | Clinical and experimental allergy |
| Il33 | 45 | 19841166 | 2009 | Journal of immunology (Baltimore, Md. : 1950) |
| Il33 | 45 | 19801525 | 2009 | Journal of immunology (Baltimore, Md. : 1950) |
| Il33 | 45 | 19763788 | 2010 | Inflammation research |
| Il33 | 45 | 19439663 | 2009 | Proceedings of the National Academy of Sciences of the United States of America |
| Il33 | 45 | 19234154 | 2009 | Journal of immunology (Baltimore, Md. : 1950) |
| Il33 | 45 | 19198610 | 2009 | Nature genetics |
| Il33 | 45 | 19064280 | 2009 | The Journal of allergy and clinical immunology |
| Il33 | 45 | 18802081 | 2008 | Journal of immunology (Baltimore, Md. : 1950) |
| Il33 | 45 | 18539196 | 2008 | The Journal of allergy and clinical immunology |
| Il33 | 45 | 17623648 | 2007 | The Journal of biological chemistry |
| Ch25h | 0 | 0 |  |  |
| Ifit3 | 0 | 0 |  |  |
| Igf1 | 1 | 16973978 | 2006 | American journal of respiratory and critical care medicine |
| Vnn1 | 0 | 0 |  |  |
| Pon1 | 5 | 22738861 | 2012 | Metabolism: clinical and experimental |
| Pon1 | 5 | 19575027 | 2009 | Journal of human genetics |
| Pon1 | 5 | 19556304 | 2009 | International immunology |
| Pon1 | 5 | 16943596 | 2006 | Biological trace element research |
| Pon1 | 5 | 15210868 | 2004 | Molecular interventions |
| Snx10 | 0 | 0 |  |  |
| Ifi30 | 0 | 0 |  |  |
| Itgb2 | 48 | 22157542 | 2012 | The Journal of nutrition |
| Itgb2 | 48 | 22004287 | 2011 | Respiratory research |
| Itgb2 | 48 | 21985360 | 2011 | Clinical and experimental immunology |
| Itgb2 | 48 | 20413544 | 2010 | The European respiratory journal |
| Itgb2 | 48 | 20351460 | 2010 | Journal of infection in developing countries |
| Itgb2 | 48 | 19463772 | 2009 | Experimental hematology |
| Itgb2 | 48 | 18771439 | 2009 | Immunology |
| Itgb2 | 48 | 18760454 | 2008 | The Journal of allergy and clinical immunology |
| Itgb2 | 48 | 18684982 | 2008 | Journal of immunology (Baltimore, Md. : 1950) |
| Itgb2 | 48 | 18653650 | 2008 | The European respiratory journal |
| Itgb2 | 48 | 18504400 | 2008 | International archives of allergy and immunology |
| Itgb2 | 48 | 18056392 | 2007 | Journal of immunology (Baltimore, Md. : 1950) |
| Itgb2 | 48 | 17379071 | 2007 | Experimental hematology |
| Itgb2 | 48 | 17052676 | 2006 | International immunopharmacology |
| Itgb2 | 48 | 16798840 | 2006 | International immunology |
| Itgb2 | 48 | 16601351 | 2006 | International archives of allergy and immunology |
| Itgb2 | 48 | 16601240 | 2006 | American journal of respiratory cell and molecular biology |
| Itgb2 | 48 | 16393658 | 2006 | Environmental health perspectives |
| Itgb2 | 48 | 12877819 | 2003 | Pulmonary pharmacology & therapeutics |
| Itgb2 | 48 | 12760968 | 2003 | American journal of respiratory cell and molecular biology |
| Itgb2 | 48 | 11504695 | 2001 | American journal of physiology. Lung cellular and molecular physiology |
| Itgb2 | 48 | 10893047 | 2000 | Inflammation research |
| Itgb2 | 48 | 10706734 | 2000 | Journal of immunology (Baltimore, Md. : 1950) |
| Itgb2 | 48 | 10651778 | 2000 | Clinical and experimental allergy |
| Itgb2 | 48 | 10453752 | 1998 | Physiological research / Academia Scientiarum Bohemoslovaca |
| Itgb2 | 48 | 10390902 | 1999 | ArerugÄ« = [Allergy] |
| Itgb2 | 48 | 10340944 | 1999 | American journal of respiratory cell and molecular biology |
| Itgb2 | 48 | 10229100 | 1999 | European journal of immunology |
| Itgb2 | 48 | 9860039 | 1998 | Annals of allergy, asthma & immunology |
| Itgb2 | 48 | 9766628 | 1998 | Journal of leukocyte biology |
| Itgb2 | 48 | 9758896 | 1998 | International archives of allergy and immunology |
| Itgb2 | 48 | 9730868 | 1998 | American journal of respiratory cell and molecular biology |
| Itgb2 | 48 | 9670977 | 1998 | Journal of immunology (Baltimore, Md. : 1950) |
| Itgb2 | 48 | 9561931 | 1998 | Inflammation |
| Itgb2 | 48 | 9561930 | 1998 | Inflammation |
| Itgb2 | 48 | 9561923 | 1998 | Inflammation |
| Itgb2 | 48 | 9187566 | 1997 | Internal medicine (Tokyo, Japan) |
| Itgb2 | 48 | 9117017 | 1997 | American journal of respiratory and critical care medicine |
| Itgb2 | 48 | 8886838 | 1996 | Human gene therapy |
| Itgb2 | 48 | 8871058 | 1996 | The European respiratory journal. Supplement |
| Itgb2 | 48 | 7576691 | 1995 | American journal of respiratory cell and molecular biology |
| Itgb2 | 48 | 7812576 | 1995 | American journal of respiratory and critical care medicine |
| Itgb2 | 48 | 7596088 | 1995 | The Kurume medical journal |
| Itgb2 | 48 | 7829126 | 1994 | Immunology letters |
| Itgb2 | 48 | 1358975 | 1992 | Journal of immunology (Baltimore, Md. : 1950) |
| Itgb2 | 48 | 1353976 | 1992 | American journal of respiratory cell and molecular biology |
| Itgb2 | 48 | 1586739 | 1992 | Blood |
| Itgb2 | 48 | 1682072 | 1991 | Clinical and experimental immunology |
| Clec4a2 | 0 | 0 |  |  |
| Clec4n | 0 | 0 |  |  |
| C1ra | 0 | 0 |  |  |
| C1rb | 0 | 0 |  |  |
| B4galnt1 | 0 | 0 |  |  |
| Capg | 0 | 0 |  |  |
| Reg3g | 0 | 0 |  |  |
| Psap | 0 | 0 |  |  |
