# Supplementary material for: Integrating Murine Gene Expression Studies to Understand Obstructive Lung Disease Due to Chronic Inhaled Endotoxin
Source: PLoS One. 2013 May 13;8(5):e62910. doi: 10.1371/journal.pone.0062910 (PMC3652821; doi:10.1371/journal.pone.0062910)
Supplement: Table S3 — Pubmed search of genes present in gene signature previously reported to be associated with COPD. (DOCX) [file pone.0062910.s006.docx]

**Supplementary Table S3.** Pubmed search of genes present in gene signature previously reported to be associated with COPD

| Gene Symbol | count | PMID | Year | Journal |
| --- | --- | --- | --- | --- |
| Tgfbi | 2 | 22617718 | 2012 | Epigenetics : official journal of the DNA Methylation Society |
| Tgfbi | 2 | 16710170 | 2006 | Molecular vision |
| Matn4 | 0 | 0 |  |  |
| Clec7a | 0 | 0 |  |  |
| Olr1 | 1 | 21412277 | 2011 | Cell death & disease |
| Ctsz | 0 | 0 |  |  |
| Mmp12 | 64 | 22952876 | 2012 | PloS one |
| Mmp12 | 64 | 22949406 | 2012 | Journal of cellular biochemistry |
| Mmp12 | 64 | 22888638 | NA | Molekuliarnaia biologiia |
| Mmp12 | 64 | 22773692 | 2012 | American journal of physiology. Lung cellular and molecular physiology |
| Mmp12 | 64 | 22305682 | 2012 | The Journal of allergy and clinical immunology |
| Mmp12 | 64 | 22209925 | 2011 | Current opinion in pulmonary medicine |
| Mmp12 | 64 | 21960547 | 2012 | American journal of respiratory cell and molecular biology |
| Mmp12 | 64 | 21784967 | 2011 | American journal of physiology. Lung cellular and molecular physiology |
| Mmp12 | 64 | 21778810 | 2011 | Allergology international : official journal of the Japanese Society of Allergology |
| Mmp12 | 64 | 21647421 | 2011 | PloS one |
| Mmp12 | 64 | 21524282 | 2011 | Respiratory research |
| Mmp12 | 64 | 21445523 | 2011 | Brazilian journal of medical and biological |
| Mmp12 | 64 | 21378275 | 2011 | Blood |
| Mmp12 | 64 | 20920189 | 2010 | Respiratory research |
| Mmp12 | 64 | 20815658 | 2010 | Experimental lung research |
| Mmp12 | 64 | 20546881 | 2010 | The Journal of allergy and clinical immunology |
| Mmp12 | 64 | 20395558 | 2010 | American journal of respiratory and critical care medicine |
| Mmp12 | 64 | 20364456 | 2010 | The New England journal of medicine |
| Mmp12 | 64 | 20357289 | 2010 | The New England journal of medicine |
| Mmp12 | 64 | 20133923 | 2010 | American journal of respiratory and critical care medicine |
| Mmp12 | 64 | 20074461 | NA | International journal of immunopathology and pharmacology |
| Mmp12 | 64 | 20018959 | 2009 | The New England journal of medicine |
| Mmp12 | 64 | 19797132 | 2010 | The European respiratory |
| Mmp12 | 64 | 19706765 | 2009 | Cancer research |
| Mmp12 | 64 | 19536155 | 2009 | Nature |
| Mmp12 | 64 | 19293200 | 2009 | Therapeutic advances in respiratory disease |
| Mmp12 | 64 | 18619044 | 2008 | Genetika |
| Mmp12 | 64 | 18334288 | 2008 | Matrix biology : journal of the International Society for Matrix Biology |
| Mmp12 | 64 | 17601747 | 2007 | Protein expression and purification |
| Mmp12 | 64 | 17132494 | 2006 | Methods in enzymology |
| Mmp12 | 64 | 16982869 | 2006 | Journal of immunology (Baltimore, Md. : 1950) |
| Mmp12 | 64 | 16717027 | 2006 | Inhalation toxicology |
| Mmp12 | 64 | 16676616 | 2005 | Zhonghua liu xing bing xue za zhi = Zhonghua liuxingbingxue zazhi |
| Mmp12 | 64 | 16500946 | 2006 | American journal of physiology. Lung cellular and molecular physiology |
| Mmp12 | 64 | 16481329 | 2006 | The Journal of biological chemistry |
| Mmp12 | 64 | 16359550 | 2005 | Respiratory research |
| Mmp12 | 64 | 16308335 | 2006 | Thorax |
| Mmp12 | 64 | 16166618 | 2005 | American journal of respiratory and critical care medicine |
| Mmp12 | 64 | 15983040 | 2005 | The Journal of biological chemistry |
| Mmp12 | 64 | 15962117 | 2005 | MemÃ³rias do Instituto Oswaldo Cruz |
| Mmp12 | 64 | 15781250 | 2005 | Biochemical and biophysical research communications |
| Mmp12 | 64 | 15723202 | 2005 | Inflammation research |
| Mmp12 | 64 | 15699789 | 2005 | Current opinion in pulmonary medicine |
| Mmp12 | 64 | 15474460 | 2004 | Biochemical and biophysical research communications |
| Mmp12 | 64 | 12851242 | 2003 | American journal of respiratory and critical care medicine |
| Mmp12 | 64 | 12684241 | 2003 | American journal of respiratory and critical care medicine |
| Mmp12 | 64 | 12634787 | 2003 | Nature |
| Mmp12 | 64 | 12634771 | 2003 | Nature |
| Mmp12 | 64 | 12504900 | 2003 | Archives of biochemistry and biophysics |
| Mmp12 | 64 | 12383023 | 2002 | American journal of pharmacogenomics |
| Mmp12 | 64 | 12225964 | 2002 | American journal of physiology. Lung cellular and molecular physiology |
| Mmp12 | 64 | 11893658 | 2002 | Chest |
| Mmp12 | 64 | 11875051 | 2002 | Human molecular genetics |
| Mmp12 | 64 | 11575929 | 2001 | Journal of molecular biology |
| Mmp12 | 64 | 11575928 | 2001 | Journal of molecular biology |
| Mmp12 | 64 | 11237688 | 2001 | Protein expression and purification |
| Mmp12 | 64 | 11199097 | 2001 | Novartis Foundation symposium |
| Mmp12 | 64 | 11133493 | 2001 | American journal of physiology. Lung cellular and molecular physiology |
| Mmp12 | 64 | 10998200 | 2000 | Proceedings of the Society for Experimental Biology and Medicine. |
| Mmp12 | 64 | 10801980 | 2000 | Proceedings of the National Academy of Sciences of the United States of America |
| Mmp12 | 64 | 10605792 | 1999 | Thrombosis and haemostasis |
| Mmp12 | 64 | 10201943 | 1999 | Journal of immunology (Baltimore, Md. : 1950) |
| Mmp12 | 64 | 9230755 | 1997 | American journal of respiratory and critical care medicine |
| Mmp12 | 64 | 2438967 | 1987 | The American review of respiratory disease |
| Per3 | 0 | 0 |  |  |
| Dab2 | 0 | 0 |  |  |
| Slc3a2 | 0 | 0 |  |  |
| Cyba | 1 | 20080081 | 2010 | Clinica chimica acta; international journal of clinical chemistry |
| Fpr2 | 1 | 22215599 | 2012 | Proceedings of the National Academy of Sciences of the United States of America |
| Ctsk | 3 | 19060845 | 2009 | Modern pathology |
| Ctsk | 3 | 17227755 | 2007 | The Journal of biological chemistry |
| Ctsk | 3 | 15161653 | 2004 | The American journal of pathology |
| Ctss | 0 | 0 |  |  |
| Dbp | 23 | 21930252 | 2011 | Respiratory physiology & neurobiology |
| Dbp | 23 | 21228423 | 2011 | Thorax |
| Dbp | 23 | 19858350 | 2009 | Chronic respiratory disease |
| Dbp | 23 | 19552093 | 2008 | Journal of the Indian Medical Association |
| Dbp | 23 | 19386071 | 2009 | Respirology (Carlton, Vic.) |
| Dbp | 23 | 19002085 | 2008 | PostÈ©py higieny i medycyny doÅ›wiadczalnej (Online) |
| Dbp | 23 | 18797740 | 2008 | Jornal brasileiro de pneumologia |
| Dbp | 23 | 18336764 | 2007 | Chinese journal of tuberculosis and respiratory diseases |
| Dbp | 23 | 17568753 | 2007 | Journal of human hypertension |
| Dbp | 23 | 17192130 | 2006 | American journal of cardiovascular drugs : drugs, devices, and other interventions |
| Dbp | 23 | 16697362 | 2006 | Clinica chimica acta; international journal of clinical chemistry |
| Dbp | 23 | 16637263 | NA | Molekuliarnaia biologiia |
| Dbp | 23 | 16579403 | NA | Indian journal of physiology and pharmacology |
| Dbp | 23 | 16117430 | 2005 | Klinicheskaia meditsina |
| Dbp | 23 | 16078956 | 2005 | Journal of the American Geriatrics Society |
| Dbp | 23 | 15245906 | 2004 | Trends in biotechnology |
| Dbp | 23 | 11219471 | 2001 | Clinical therapeutics |
| Dbp | 23 | 10759446 | 2000 | The European respiratory journal |
| Dbp | 23 | 7841973 | 1994 | Monaldi archives for chest disease |
| Dbp | 23 | 1402344 | 1992 | The Journal of the Kentucky Medical Association |
| Dbp | 23 | 1616198 | 1992 | Anales espaÃ±oles de pediatrÃ­a |
| Dbp | 23 | 1982059 | 1990 | Cardiovascular drugs and therapy |
| Dbp | 23 | 2879733 | 1986 | European journal of clinical pharmacology |
| Ctsb | 0 | 0 |  |  |
| Laptm5 | 0 | 0 |  |  |
| Cxcl2 | 24 | 22360706 | 2012 | Free radical research |
| Cxcl2 | 24 | 21961642 | 2011 | Journal of environmental science and health. |
| Cxcl2 | 24 | 20887783 | 2010 | Free radical biology & medicine |
| Cxcl2 | 24 | 20818377 | 2010 | Nature medicine |
| Cxcl2 | 24 | 19744573 | 2009 | Pulmonary pharmacology & therapeutics |
| Cxcl2 | 24 | 19293939 | 2009 | PloS one |
| Cxcl2 | 24 | 19254149 | 2009 | American journal of veterinary research |
| Cxcl2 | 24 | 19050257 | 2008 | Journal of immunology (Baltimore, Md. : 1950) |
| Cxcl2 | 24 | 19004925 | 2009 | The Journal of pharmacology and experimental therapeutics |
| Cxcl2 | 24 | 18310229 | 2008 | American journal of physiology. Lung cellular and molecular physiology |
| Cxcl2 | 24 | 18256171 | 2008 | The Journal of pharmacology and experimental therapeutics |
| Cxcl2 | 24 | 18052742 | 2007 | American journal of veterinary research |
| Cxcl2 | 24 | 18021431 | 2007 | Respiratory research |
| Cxcl2 | 24 | 18007984 | 2007 | Environmental health perspectives |
| Cxcl2 | 24 | 17766584 | 2007 | American journal of physiology. Lung cellular and molecular physiology |
| Cxcl2 | 24 | 17690174 | 2007 | American journal of physiology. Gastrointestinal and liver physiology |
| Cxcl2 | 24 | 16929007 | 2006 | Toxicological sciences : an official journal of the Society of Toxicology |
| Cxcl2 | 24 | 15833762 | 2005 | American journal of physiology. Lung cellular and molecular physiology |
| Cxcl2 | 24 | 15668323 | 2005 | American journal of respiratory cell and molecular biology |
| Cxcl2 | 24 | 15333327 | 2004 | American journal of respiratory cell and molecular biology |
| Cxcl2 | 24 | 12476359 | 2003 | Inhalation toxicology |
| Cxcl2 | 24 | 12359653 | 2002 | American journal of respiratory and critical care medicine |
| Cxcl2 | 24 | 11798689 | 1999 | Zhonghua nei ke za zhi [Chinese journal of internal medicine] |
| Cxcl2 | 24 | 9847020 | 1998 | Veterinary immunology and immunopathology |
| Saa3 | 0 | 0 |  |  |
| Tmem106a | 0 | 0 |  |  |
| Prkcd | 0 | 0 |  |  |
| Clu | 0 | 0 |  |  |
| Smpdl3b | 0 | 0 |  |  |
| Lair1 | 0 | 0 |  |  |
| Itih4 | 1 | 18618493 | 2008 | Proteomics |
| Grn | 0 | 0 |  |  |
| Tgfbr1 | 0 | 0 |  |  |
| Lrg1 | 0 | 0 |  |  |
| Cd1d1 | 0 | 0 |  |  |
| C3 | 57 | 22462235 | 2011 | Journal of traditional Chinese medicine |
| C3 | 57 | 21846943 | 2011 | Disease markers |
| C3 | 57 | 21813741 | 2011 | Radiology |
| C3 | 57 | 21524765 | 2011 | Medicina clÃ­nica |
| C3 | 57 | 21270401 | 2011 | Journal of immunology (Baltimore, Md. : 1950) |
| C3 | 57 | 20144890 | 2010 | Experimental neurology |
| C3 | 57 | 19922730 | 2009 | Current rheumatology reports |
| C3 | 57 | 19684087 | 2009 | Journal of immunology (Baltimore, Md. : 1950) |
| C3 | 57 | 19101763 | NA | Marine biotechnology (New York, N.Y.) |
| C3 | 57 | 18403672 | 2008 | Chest |
| C3 | 57 | 17975205 | 2008 | American journal of respiratory and critical care medicine |
| C3 | 57 | 17502296 | NA | Annales de biologie clinique |
| C3 | 57 | 17471436 | 2007 | The Journal of infectious diseases |
| C3 | 57 | 17331971 | 2007 | The European respiratory journal |
| C3 | 57 | 16966403 | 2006 | Infection and immunity |
| C3 | 57 | 16711502 | 2006 | Revista espaÃ±ola de anestesiologÃ­a y reanimaciÃ³n |
| C3 | 57 | 16574942 | 2006 | American journal of respiratory cell and molecular biology |
| C3 | 57 | 16571611 | 2006 | The European respiratory journal |
| C3 | 57 | 16512391 | 2006 | Klinicheskaia meditsina |
| C3 | 57 | 16113417 | 2004 | Proceedings of the American Thoracic Society |
| C3 | 57 | 15159749 | 2004 | StomatologiiÍ¡a |
| C3 | 57 | 14563253 | 2003 | Revista clÃ­nica espaÃ±ola |
| C3 | 57 | 12615868 | 2003 | The Journal of antimicrobial chemotherapy |
| C3 | 57 | 11606842 | 2001 | Investigative radiology |
| C3 | 57 | 11591733 | 2001 | Journal of immunology (Baltimore, Md. : 1950) |
| C3 | 57 | 11371519 | 2001 | Journal of bacteriology |
| C3 | 57 | 11045117 | 2000 | Presse mÃ©dicale (Paris, France : 1983) |
| C3 | 57 | 10218320 | 1998 | Journal of the Indian Medical Association |
| C3 | 57 | 10189505 | 1999 | Acta neurochirurgica |
| C3 | 57 | 9266867 | 1997 | Chest |
| C3 | 57 | 9071161 | 1997 | Nihon KyÅbu Shikkan Gakkai zasshi |
| C3 | 57 | 8926173 | 1996 | Der Hautarzt |
| C3 | 57 | 8541823 | 1995 | Monaldi archives for chest disease |
| C3 | 57 | 7751050 | 1995 | The Indian journal of medical research |
| C3 | 57 | 7878554 | 1994 | Thorax |
| C3 | 57 | 8137654 | 1993 | Zhonghua nei ke za zhi [Chinese journal of internal medicine] |
| C3 | 57 | 1564151 | 1992 | Journal of the American Academy of Dermatology |
| C3 | 57 | 2228068 | 1990 | The Indian journal of medical research |
| C3 | 57 | 2129476 | NA | Archivos de investigaciÃ³n mÃ©dica |
| C3 | 57 | 2196033 | 1990 | Archives of virology |
| C3 | 57 | 2784010 | 1989 | Stereotactic and functional neurosurgery |
| C3 | 57 | 3069024 | 1988 | Annales de dermatologie et de vÃ©nÃ©rÃ©ologie |
| C3 | 57 | 3649280 | 1987 | Clinical immunology and immunopathology |
| C3 | 57 | 3454206 | 1987 | Acta paediatrica Hungarica |
| C3 | 57 | 3702213 | 1986 | Kidney international |
| C3 | 57 | 2424179 | 1986 | VÅ­treshni bolesti |
| C3 | 57 | 3875903 | 1985 | South African medical journal |
| C3 | 57 | 6208579 | NA | Revista clÃ­nica espaÃ±ola |
| C3 | 57 | 6978407 | 1982 | Journal of clinical & laboratory immunology |
| C3 | 57 | 7251858 | 1981 | The Journal of clinical investigation |
| C3 | 57 | 6911988 | 1981 | Acta medica Austriaca |
| C3 | 57 | 7202596 | 1980 | European journal of respiratory diseases |
| C3 | 57 | 7354236 | 1980 | The Journal of laboratory and clinical medicine |
| C3 | 57 | 112044 | 1979 | Immunology |
| C3 | 57 | 308809 | 1978 | British journal of diseases of the chest |
| C3 | 57 | 1004644 | 1976 | Naunyn-Schmiedeberg's archives of pharmacology |
| C3 | 57 | 806400 | 1975 | Clinical allergy |
| LOC100048759 | 0 | 0 |  |  |
| Fn1 | 0 | 0 |  |  |
| Emr1 | 0 | 0 |  |  |
| Chi3l3 | 1 | 21915293 | 2011 | PloS one |
| Acp2 | 0 | 0 |  |  |
| H2-Ab1 | 0 | 0 |  |  |
| Havcr2 | 0 | 0 |  |  |
| Cfb | 1 | 21697997 | 2011 | International journal of chronic obstructive pulmonary disease |
| C2 | 24 | 22462235 | 2011 | Journal of traditional Chinese medicine |
| C2 | 24 | 22458856 | 2012 | Journal of environmental science and health |
| C2 | 24 | 21813741 | 2011 | Radiology |
| C2 | 24 | 19922730 | 2009 | Current rheumatology reports |
| C2 | 24 | 19716045 | 2009 | The Journal of heart and lung transplantation |
| C2 | 24 | 19651244 | 2009 | Respiratory physiology & neurobiology |
| C2 | 24 | 19136241 | 2009 | Respiratory medicine |
| C2 | 24 | 17804442 | 2008 | The European respiratory journal |
| C2 | 24 | 17384086 | 2007 | American journal of physiology. Lung cellular and molecular physiology |
| C2 | 24 | 17379851 | 2007 | American journal of respiratory and critical care medicine |
| C2 | 24 | 16102443 | 2005 | The Journal of heart and lung transplantation |
| C2 | 24 | 16008605 | 2005 | Clinical transplantation |
| C2 | 24 | 15171561 | 2004 | Zhongguo yi xue ke xue yuan xue bao. Acta Academiae Medicinae Sinicae |
| C2 | 24 | 12615868 | 2003 | The Journal of antimicrobial chemotherapy |
| C2 | 24 | 12561617 | 2001 | Wei sheng yan jiu = Journal of hygiene research |
| C2 | 24 | 12184862 | 2002 | Journal of aerosol medicine |
| C2 | 24 | 11964752 | 2002 | Current opinion in allergy and clinical immunology |
| C2 | 24 | 11561763 | 2001 | Virchows Archiv : an international journal of pathology |
| C2 | 24 | 10780759 | 2000 | The European respiratory journal |
| C2 | 24 | 9641386 | 1998 | The European journal of surgery. |
| C2 | 24 | 3565938 | 1987 | The American review of respiratory disease |
| C2 | 24 | 3773900 | 1986 | Monographs in allergy |
| C2 | 24 | 6836186 | 1983 | Research in veterinary science |
| C2 | 24 | 663425 | 1978 | Respiration; international review of thoracic diseases |
| Cp | 72 | 23132203 | 2012 | Medical care |
| Cp | 72 | 23000935 | 2012 | The American journal of geriatric psychiatry |
| Cp | 72 | 22726610 | 2012 | BMC pulmonary medicine |
| Cp | 72 | 21439045 | 2011 | Biomedical engineering online |
| Cp | 72 | 21340182 | NA | Brazilian journal of otorhinolaryngology |
| Cp | 72 | 21211434 | 2010 | Chinese journal of stomatology |
| Cp | 72 | 21157643 | 2010 | Current opinion in investigational drugs (London, England : 2000) |
| Cp | 72 | 21110197 | 2011 | Heart and vessels |
| Cp | 72 | 21092098 | 2010 | Trials |
| Cp | 72 | 20536425 | 2010 | Current topics in medicinal chemistry |
| Cp | 72 | 20463253 | 2010 | Proceedings of the American Thoracic Society |
| Cp | 72 | 19998041 | 2010 | Lung |
| Cp | 72 | 19995653 | 2009 | Revue de pneumologie clinique |
| Cp | 72 | 19560768 | 2009 | Gastrointestinal endoscopy |
| Cp | 72 | 19350630 | 2009 | International journal of cancer. Journal international du cancer |
| Cp | 72 | 19058490 | NA | Revista alergia Mexico (Tecamachalco, Puebla, Mexico : 1993) |
| Cp | 72 | 19052510 | 2008 | ArerugÄ« = [Allergy] |
| Cp | 72 | 18619824 | 2008 | Respiratory medicine |
| Cp | 72 | 18441096 | 2008 | American journal of physiology. Lung cellular and molecular physiology |
| Cp | 72 | 18188083 | 2008 | Journal of occupational and environmental medicine |
| Cp | 72 | 18028276 | 2008 | Transfusion |
| Cp | 72 | 17526197 | 2007 | TerapevticheskiÄ­ arkhiv |
| Cp | 72 | 17287299 | 2007 | Thorax |
| Cp | 72 | 16495069 | 2006 | European journal of cardio-thoracic surgery |
| Cp | 72 | 16249920 | 2006 | European journal of applied physiology |
| Cp | 72 | 16249313 | 2005 | Physiological genomics |
| Cp | 72 | 16236083 | 2005 | International journal of clinical practice |
| Cp | 72 | 16222887 | NA | The Journal of international medical research |
| Cp | 72 | 16113464 | 2005 | Proceedings of the American Thoracic Society |
| Cp | 72 | 16002925 | 2005 | Chest |
| Cp | 72 | 15474975 | 2005 | The international journal of biochemistry & cell biology |
| Cp | 72 | 15289103 | 2004 | Journal of molecular biology |
| Cp | 72 | 14769726 | 2004 | Chest |
| Cp | 72 | 14512671 | NA | Respiration; international review of thoracic diseases |
| Cp | 72 | 12740284 | 2003 | Chest |
| Cp | 72 | 12504900 | 2003 | Archives of biochemistry and biophysics |
| Cp | 72 | 12371533 | 2002 | Journal of investigational allergology & clinical immunology |
| Cp | 72 | 12211408 | 2002 | Current opinion in investigational drugs (London, England : 2000) |
| Cp | 72 | 11980276 | 2001 | Monaldi archives for chest disease |
| Cp | 72 | 11798603 | 2001 | Zhonghua nei ke za zhi [Chinese journal of internal medicine] |
| Cp | 72 | 11780351 | 2001 | Chinese medical journal |
| Cp | 72 | 11171871 | 2001 | International journal of epidemiology |
| Cp | 72 | 11075875 | 2000 | Cancer causes & control : CCC |
| Cp | 72 | 10934068 | 2000 | American journal of respiratory and critical care medicine |
| Cp | 72 | 10232436 | 1999 | The European respiratory journal |
| Cp | 72 | 10193378 | 1998 | Thorax |
| Cp | 72 | 9253724 | 1997 | Journal of occupational and environmental medicine |
| Cp | 72 | 7593895 | 1995 | Intensive care medicine |
| Cp | 72 | 7647736 | 1995 | Pneumonologia i alergologia polska |
| Cp | 72 | 8005246 | 1994 | The European respiratory journal |
| Cp | 72 | 8310540 | 1993 | La Tunisie mÃ©dicale |
| Cp | 72 | 8368923 | 1993 | Archives of surgery (Chicago, Ill. : 1960) |
| Cp | 72 | 8511687 | 1993 | South African medical journal |
| Cp | 72 | 1339048 | NA | Cancer epidemiology, biomarkers & prevention |
| Cp | 72 | 1585224 | 1992 | South African medical journal |
| Cp | 72 | 1392658 | 1992 | Polish journal of occupational medicine and environmental health |
| Cp | 72 | 1895584 | 1991 | Nihon KyÅbu Shikkan Gakkai zasshi |
| Cp | 72 | 2129991 | 1990 | Chirurgie |
| Cp | 72 | 2804252 | NA | Biopharmaceutics & drug disposition |
| Cp | 72 | 2805949 | 1989 | Zhonghua nei ke za zhi [Chinese journal of internal medicine] |
| Cp | 72 | 2919340 | 1989 | South African medical journal |
| Cp | 72 | 2688602 | 1989 | Arquivos brasileiros de cardiologia |
| Cp | 72 | 3258445 | 1988 | South African medical journal |
| Cp | 72 | 3356633 | 1988 | Journal of applied physiology (Bethesda, Md. : 1985) |
| Cp | 72 | 3622017 | 1987 | Chest |
| Cp | 72 | 4048084 | 1985 | Preventive medicine |
| Cp | 72 | 6508357 | NA | Archives of environmental health |
| Cp | 72 | 6838047 | 1983 | The American review of respiratory disease |
| Cp | 72 | 6837720 | 1983 | The American journal of pathology |
| Cp | 72 | 7065516 | 1982 | The American review of respiratory disease |
| Cp | 72 | 7330656 | 1981 | Schweizerische medizinische Wochenschrift |
| Cp | 72 | 760740 | 1979 | British journal of clinical pharmacology |
| Ly6i | 0 | 0 |  |  |
| Pigr | 7 | 22053820 | 2012 | Journal of proteome research |
| Pigr | 7 | 21512171 | 2011 | American journal of respiratory and critical care medicine |
| Pigr | 7 | 20706611 | 2010 | Journal of biomedicine & biotechnology |
| Pigr | 7 | 12654638 | 2003 | American journal of respiratory cell and molecular biology |
| Pigr | 7 | 12615618 | 2003 | American journal of respiratory and critical care medicine |
| Pigr | 7 | 11208645 | 2001 | American journal of respiratory and critical care medicine |
| Pigr | 7 | 11082760 | 2000 | Acta oto-rhino-laryngologica Belgica |
| C1qb | 0 | 0 |  |  |
| Bst1 | 0 | 0 |  |  |
| Muc1 | 13 | 21569324 | 2011 | BMC pulmonary medicine |
| Muc1 | 13 | 21474912 | 2011 | Respiration; international review of thoracic diseases |
| Muc1 | 13 | 21206098 | 2011 | JOP : Journal of the pancreas |
| Muc1 | 13 | 20886351 | 2011 | International archives of occupational and environmental health |
| Muc1 | 13 | 20538446 | 2010 | Respiratory medicine |
| Muc1 | 13 | 19960788 | 2009 | Sarcoidosis, vasculitis, and diffuse lung diseases |
| Muc1 | 13 | 18595202 | 2008 | Biomarkers : biochemical indicators of exposure, response, and susceptibility to chemicals |
| Muc1 | 13 | 16969297 | 2006 | Transplantation |
| Muc1 | 13 | 12605318 | 2003 | Clinical rheumatology |
| Muc1 | 13 | 12010847 | 2002 | Chest |
| Muc1 | 13 | 11015008 | 2000 | Nephron |
| Muc1 | 13 | 9685530 | 1998 | Lung |
| Muc1 | 13 | 7548906 | 1995 | Tubercle and lung disease |
| Lgals3bp | 0 | 0 |  |  |
| Hvcn1 | 0 | 0 |  |  |
| Slc6a20a | 0 | 0 |  |  |
| Orm1 | 0 | 0 |  |  |
| Orm2 | 0 | 0 |  |  |
| Bcl2a1d | 0 | 0 |  |  |
| Bcl2a1a | 0 | 0 |  |  |
| Bcl2a1b | 0 | 0 |  |  |
| Csf2rb2 | 0 | 0 |  |  |
| Tifa | 0 | 0 |  |  |
| Itgax | 4 | 16940747 | 2006 | International archives of allergy and immunology |
| Itgax | 4 | 16907910 | 2006 | Clinical and experimental immunology |
| Itgax | 4 | 16424380 | 2006 | American journal of respiratory cell and molecular biology |
| Itgax | 4 | 8902456 | 1996 | The European respiratory journal |
| Il1rn | 15 | 23071879 | 2012 | Oman medical journal |
| Il1rn | 15 | 22343222 | 2012 | American journal of respiratory cell and molecular biology |
| Il1rn | 15 | 22163019 | 2011 | PloS one |
| Il1rn | 15 | 21814463 | 2011 | International journal of chronic obstructive pulmonary disease |
| Il1rn | 15 | 20650986 | 2011 | The European respiratory journal |
| Il1rn | 15 | 20064207 | 2010 | Arthritis research & therapy |
| Il1rn | 15 | 19608716 | 2009 | American journal of respiratory and critical care medicine |
| Il1rn | 15 | 19291375 | 2009 | Journal of clinical immunology |
| Il1rn | 15 | 18579366 | 2008 | Respiratory medicine |
| Il1rn | 15 | 18364273 | 2008 | The Kaohsiung journal of medical sciences |
| Il1rn | 15 | 17380888 | NA | Molekuliarnaia biologiia |
| Il1rn | 15 | 15766560 | 2005 | Biochemical and biophysical research communications |
| Il1rn | 15 | 12928941 | 2003 | Zeitschrift fÃ¼r Rheumatologie |
| Il1rn | 15 | 12467523 | 2002 | Mediators of inflammation |
| Il1rn | 15 | 11053025 | 2000 | American journal of physiology. Lung cellular and molecular physiology |
| Chi3l1 | 7 | 22554524 | 2012 | Biochemical and biophysical research communications |
| Chi3l1 | 7 | 21968467 | 2012 | Respiration; international review of thoracic diseases |
| Chi3l1 | 7 | 21949714 | 2011 | PloS one |
| Chi3l1 | 7 | 21915293 | 2011 | PloS one |
| Chi3l1 | 7 | 20656949 | 2011 | American journal of respiratory cell and molecular biology |
| Chi3l1 | 7 | 19491341 | 2009 | American journal of respiratory cell and molecular biology |
| Chi3l1 | 7 | 18802121 | 2008 | Journal of immunology (Baltimore, Md. : 1950) |
| Cd68 | 38 | 22798194 | 2012 | Cell biochemistry and biophysics |
| Cd68 | 38 | 22362876 | 2012 | Chest |
| Cd68 | 38 | 22215599 | 2012 | Proceedings of the National Academy of Sciences of the United States of America |
| Cd68 | 38 | 21970519 | 2011 | Respiratory research |
| Cd68 | 38 | 21681974 | 2011 | Diagnostic cytopathology |
| Cd68 | 38 | 21512269 | 2011 | Experimental animals / Japanese Association for Laboratory Animal Science |
| Cd68 | 38 | 21197447 | 2010 | Mediators of inflammation |
| Cd68 | 38 | 20495756 | 2010 | Romanian journal of morphology and embryology |
| Cd68 | 38 | 20472710 | 2010 | American journal of physiology. Lung cellular and molecular physiology |
| Cd68 | 38 | 19736178 | 2009 | Thorax |
| Cd68 | 38 | 19353346 | 2008 | COPD |
| Cd68 | 38 | 19218194 | 2009 | American journal of respiratory and critical care medicine |
| Cd68 | 38 | 19118262 | 2009 | Chest |
| Cd68 | 38 | 18268925 | 2007 | International journal of chronic obstructive pulmonary disease |
| Cd68 | 38 | 19105585 | 2008 | Drugs |
| Cd68 | 38 | 17988392 | 2007 | Respiratory research |
| Cd68 | 38 | 17557771 | 2007 | Thorax |
| Cd68 | 38 | 17504799 | 2007 | The European respiratory journal |
| Cd68 | 38 | 16424444 | 2006 | American journal of respiratory and critical care medicine |
| Cd68 | 38 | 16050471 | 2005 | journal of the Japanese Respiratory Society |
| Cd68 | 38 | 20477652 | 2005 | Expert review of clinical immunology |
| Cd68 | 38 | 15607122 | 2005 | Pulmonary pharmacology & therapeutics |
| Cd68 | 38 | 15047949 | 2004 | Thorax |
| Cd68 | 38 | 14605067 | 2003 | Chest |
| Cd68 | 38 | 12816740 | 2003 | American journal of respiratory and critical care medicine |
| Cd68 | 38 | 12668802 | 2003 | Thorax |
| Cd68 | 38 | 12149529 | 2002 | Thorax |
| Cd68 | 38 | 12070058 | 2002 | American journal of respiratory and critical care medicine |
| Cd68 | 38 | 11199094 | 2001 | Novartis Foundation symposium |
| Cd68 | 38 | 10984367 | 2000 | The Journal of allergy and clinical immunology |
| Cd68 | 38 | 10843939 | 2000 | Chest |
| Cd68 | 38 | 10802223 | 2000 | Free radical biology & medicine |
| Cd68 | 38 | 10607796 | 2000 | Thorax |
| Cd68 | 38 | 10435362 | 1999 | Internal medicine (Tokyo, Japan) |
| Cd68 | 38 | 9847291 | 1998 | American journal of respiratory and critical care medicine |
| Cd68 | 38 | 9403729 | 1997 | The American journal of pathology |
| Cd68 | 38 | 9117016 | 1997 | American journal of respiratory and critical care medicine |
| Cd68 | 38 | 8564109 | 1996 | American journal of respiratory and critical care medicine |
| Gatm | 0 | 0 |  |  |
| Olfm1 | 0 | 0 |  |  |
| Sirpa | 0 | 0 |  |  |
| Ptgs1 | 6 | 22324934 | 2012 | Expert opinion on therapeutic targets |
| Ptgs1 | 6 | 22204820 | 2012 | Immunobiology |
| Ptgs1 | 6 | 21798652 | 2011 | Archivos de bronconeumologÃ­a |
| Ptgs1 | 6 | 21458581 | 2011 | Prostaglandins & other lipid mediators |
| Ptgs1 | 6 | 15921208 | NA | American journal of rhinology |
| Ptgs1 | 6 | 15301300 | 2004 | Journal of investigational allergology & clinical immunology |
| Ccl9 | 0 | 0 |  |  |
| Ccl6 | 1 | 16645178 | 2006 | American journal of respiratory cell and molecular biology |
| 1100001G20Rik | 0 | 0 |  |  |
| Bpifb1 | 0 | 0 |  |  |
| Procr | 0 | 0 |  |  |
| Lbp | 10 | 20129855 | 2010 | IEEE transactions on medical imaging |
| Lbp | 10 | 19718433 | 2009 | PloS one |
| Lbp | 10 | 19010986 | 2009 | The European respiratory journal |
| Lbp | 10 | 18979835 | 2008 | Medical image computing and computer-assisted intervention |
| Lbp | 10 | 16740168 | 2006 | BMC pulmonary medicine |
| Lbp | 10 | 15356561 | 2004 | The Journal of allergy and clinical immunology |
| Lbp | 10 | 11514694 | 2001 | Thorax |
| Lbp | 10 | 10468134 | 1999 | The Journal of infection |
| Lbp | 10 | 9731007 | 1998 | American journal of respiratory and critical care medicine |
| Lbp | 10 | 8795671 | 1996 | Thorax |
| Rab20 | 0 | 0 |  |  |
| F10 | 2 | 22970026 | 2012 | Experimental and therapeutic medicine |
| F10 | 2 | 18534165 | 2008 | Ugeskrift for laeger |
| Naip2 | 0 | 0 |  |  |
| Cd14 | 31 | 23117214 | 2013 | Biomaterials |
| Cd14 | 31 | 22355383 | 2012 | PloS one |
| Cd14 | 31 | 21439805 | 2011 | Respiratory medicine |
| Cd14 | 31 | 21129004 | 2011 | Scandinavian journal of immunology |
| Cd14 | 31 | 20709824 | 2011 | American journal of respiratory and critical care medicine |
| Cd14 | 31 | 20438701 | 2010 | Biochemical and biophysical research communications |
| Cd14 | 31 | 20080799 | 2010 | Proceedings of the National Academy of Sciences of the United States of America |
| Cd14 | 31 | 19675120 | 2010 | Innate immunity |
| Cd14 | 31 | 19361972 | 2009 | Respiratory medicine |
| Cd14 | 31 | 19119705 | 2008 | Annals of allergy, asthma & immunology |
| Cd14 | 31 | 19085563 | 2008 | Experimental lung research |
| Cd14 | 31 | 19080469 | 2008 | Zhonghua yi xue za zhi |
| Cd14 | 31 | 19010986 | 2009 | The European respiratory journal |
| Cd14 | 31 | 18446588 | 2008 | The Journal of asthma : official journal of the Association for the Care of Asthma |
| Cd14 | 31 | 17574828 | 2007 | Respiratory medicine |
| Cd14 | 31 | 17384086 | 2007 | American journal of physiology. Lung cellular and molecular physiology |
| Cd14 | 31 | 17072032 | 2006 | Journal of physiology and pharmacology |
| Cd14 | 31 | 16907910 | 2006 | Clinical and experimental immunology |
| Cd14 | 31 | 16606450 | 2006 | Respiratory research |
| Cd14 | 31 | 16406722 | 2007 | Pulmonary pharmacology & therapeutics |
| Cd14 | 31 | 16004610 | 2005 | Respiratory research |
| Cd14 | 31 | 15879152 | 2005 | Journal of immunology (Baltimore, Md. : 1950) |
| Cd14 | 31 | 15802338 | 2005 | The European respiratory journal : |
| Cd14 | 31 | 15660518 | 2005 | Annual review of medicine |
| Cd14 | 31 | 15544629 | 2004 | Clinical and experimental immunology |
| Cd14 | 31 | 15138625 | 2004 | International journal of molecular medicine |
| Cd14 | 31 | 12684293 | 2003 | Chest |
| Cd14 | 31 | 11440642 | 2001 | Journal of interferon & cytokine research |
| Cd14 | 31 | 10707942 | 2000 | Journal of medical microbiology |
| Cd14 | 31 | 8998075 | 1997 | American journal of respiratory cell and molecular biology |
| Cd14 | 31 | 8033141 | 1994 | Cancer research |
| Cd200r1 | 0 | 0 |  |  |
| Mtm1 | 0 | 0 |  |  |
| Tlr7 | 0 | 0 |  |  |
| Cybb | 4 | 18952568 | 2009 | American journal of respiratory cell and molecular biology |
| Cybb | 4 | 18403597 | 2008 | The American journal of pathology |
| Cybb | 4 | 16123991 | 2006 | Pediatric blood & cancer |
| Cybb | 4 | 15983040 | 2005 | The Journal of biological chemistry |
| Atp6ap2 | 0 | 0 |  |  |
| Id2 | 1 | 17395785 | 2007 | American journal of physiology. Regulatory, integrative and comparative physiology |
| Slc26a4 | 5 | 22116372 | 2011 | Cellular physiology and biochemistry |
| Slc26a4 | 5 | 22116359 | 2011 | Cellular physiology and biochemistry |
| Slc26a4 | 5 | 22116352 | 2011 | Cellular physiology and biochemistry |
| Slc26a4 | 5 | 21814192 | 2011 | Clinical pharmacology and therapeutics |
| Slc26a4 | 5 | 18424749 | 2008 | Journal of immunology (Baltimore, Md. : 1950) |
| Ms4a7 | 0 | 0 |  |  |
| Ms4a6d | 0 | 0 |  |  |
| Rab32 | 0 | 0 |  |  |
| Il33 | 2 | 21682745 | 2011 | Immunological reviews |
| Il33 | 2 | 20608085 | 2010 | Nihon KokyÅ«ki Gakkai zasshi = the journal of the Japanese Respiratory Society |
| Ch25h | 0 | 0 |  |  |
| Ifit3 | 0 | 0 |  |  |
| Igf1 | 0 | 0 |  |  |
| Vnn1 | 0 | 0 |  |  |
| Pon1 | 6 | 22738861 | 2012 | Metabolism: clinical and experimental |
| Pon1 | 6 | 22528954 | 2012 | Sleep & breathing = Schlaf & Atmung |
| Pon1 | 6 | 22015083 | 2011 | Respiratory medicine |
| Pon1 | 6 | 18635682 | 2008 | Journal of medical genetics |
| Pon1 | 6 | 17613085 | 2007 | Inhalation toxicology |
| Pon1 | 6 | 16380766 | 2005 | Saudi medical journal |
| Snx10 | 0 | 0 |  |  |
| Ifi30 | 0 | 0 |  |  |
| Itgb2 | 10 | 21976223 | 2011 | Clinical and vaccine immunology : CVI |
| Itgb2 | 10 | 21651795 | 2011 | Respiratory research |
| Itgb2 | 10 | 19574534 | 2010 | American journal of respiratory cell and molecular biology |
| Itgb2 | 10 | 17626109 | 2007 | The European respiratory journal |
| Itgb2 | 10 | 17573488 | 2007 | Chest |
| Itgb2 | 10 | 16807266 | 2006 | The European respiratory journal |
| Itgb2 | 10 | 11953106 | 2002 | Chinese journal of tuberculosis and respiratory diseases |
| Itgb2 | 10 | 11817553 | 2002 | Equine veterinary journal |
| Itgb2 | 10 | 10707942 | 2000 | Journal of medical microbiology |
| Itgb2 | 10 | 8902456 | 1996 | The European respiratory journal |
| Clec4a2 | 0 | 0 |  |  |
| Clec4n | 0 | 0 |  |  |
| C1ra | 0 | 0 |  |  |
| C1rb | 0 | 0 |  |  |
| B4galnt1 | 0 | 0 |  |  |
| Capg | 0 | 0 |  |  |
| Reg3g | 0 | 0 |  |  |
| Psap | 0 | 0 |  |  |

.
